# Supplementary material for: Targeting Multicopy Prophage Genes for the Increased Detection of Borrelia burgdorferi Sensu Lato (s.l.), the Causative Agents of Lyme Disease, in Blood
Source: Front Microbiol. 2021 Mar 15;12:651217. doi: 10.3389/fmicb.2021.651217 (PMC8005754; doi:10.3389/fmicb.2021.651217)
Supplement: Supplementary file 1 [file Data_Sheet_1.docx]

**Supplementary information 1: *Borrelia burgdorferi* B31 phage terminase large subunit (*terL*) nucleotide sequences**

Within the *Borrelia burgdorferi* B31 genome, there are 10 copies of the *terL* gene homologs that are identified from seven circular plasmids (cp32 serials) and three linear plasmids (lp56, lp54 and lp28-2). Eight out of 10 *terL* genes, except those located on lp54 and lp28-2, are highly similar to each other as revealed by alignment below.

**>CP32-1** - **phage terminase large subunit – NC_000948.1 (29398..30750)**

GTGAACTTATATCAAACAAAACTTTTTACAACACTACAAAAGGAATACAAAAATAAATATGGAGTTGATATATCACAATTTGTAAAGCTAACAAATTCTTCAATTAATTTTGATAAGTTTGAAGAAGAACAGTTAACTTTAAAACAAAAAAATGTGATAAAAAGCATTAAAAAGAATAATGAAAAGAAGATTATACTCAGCGGAGGCATAGCTAGTGGCAAAACGTATCTTGCATGTTATCTTTTTCTAAAAAGTTTAATTGAAATTAAAAAGTTATACTCTAGTGATACTAATAATTTCATTATAGGGAATTCACAACGTTCAGTTGAAGTTAATGTTTTGGGGCAATTTGAAAAGCTATGTAAACTTCTTAAAATTCCTTATATTCCAAGACATACAAATAATTCATATATTCTGATTGATTCACTACGTATTAATCTATATGGAGGAGATAAGGCAAGTGATTTTGAAAGATTTAGGGGAAGTAATTCGGCACTTATTTTTGTTAATGAGGCTACAACTTTACACAAGCAAACTTTAGAGGAAGTCTTAAAAAGACTAAGATGCGGGCAAGAAACTATTATTTTTGATACTAATCCTGATCATCCAGAACACTATTTTAAAACCGATTATATTGATAATATAGCGACCTTTAAGACATATAAGTTTACAACTTATGATAATGTGCTACTTAGTAAAGGATTTGTCGAAACACAAGAAAAGCTATATAAAGATATACCATCATATAAAGCAAGAGTTTTGTTAGGTGAGTGGATAGCAAGCACTGATTCAATTTTTACACAAATAAATATTACTGATGATTATGTATTTACTAGCCCGATAGCATATTTAGACCCAGCATTTAGTGTTGGCGGGGATAACACTGCATTATGTGTTATGGAGCGAGTTGATGATAAGTATTATGCTTTTGTATTTCAAGACCAAAGACCAGCTAATGATCCTTATATTATGAATATGGTAAAGACTGTTATAGAAAATTTCAATGTGCATACACTGTATTTAGAGGATAGAGATAATACAAAAGGTGCTGGTGGATTGACCCGTGAATACATCTTGCTAAGAAGTAATATAAGCCAATATTTTAGAATTGTTCCAGTTAAGCCAAAGTCTAATAAATTTAGCAGAATAACAACGTTAATTACGCCGTTTACTTACAAAAAACTTTATATTACAAAGTACAGTAGTTCTTCCGTATTTAATGATATTTATTCGTATAAGGGGGATAATAAAACCCATGATGACGCTCTTGATGCAATATCTGCAGCATATTTGATGTTGTCTTTAGGATATAGAGAGCGAAGTGTTCACTTTGGCAATCAAAGATTTTTGTAA

**>CP32-3** - **phage terminase large subunit – 28871-30223**

GTGAACTTATATCAAACAAAACTTTTTACAACACTACAAAAGGAATACAAAAATAAATATGGAGTTGATATATCACAATTTGTAAAGCTAACAAATTCTTCAATTAATTTTGATAAGTTTGAAGAAAAACAGTTAACTTTAAAACAAAAAAATGTGATAAAAAGTATTAAAAAGAATAATGAAAAAAAGATTATATTCAGCGGCGGCATAGCTAGCGGCAAAACGTATCTTGCATGCTATCTTTTTCTCAAAAGTTTAATTGAAAATAAAAAGTTATATTCTAGCGATACGAATAATTTTATTATTGGGAATTCACAACGCTCAGTTGAAGTTAATGTTTTGGGACAATTTGAAAAGCTATGTAAACTTCTTAAAATTCCTTATATTCCAAGACATACAAATAATTCATATATTCTGATTGATTCACTACGTATTAATCTATATGGTGGAGATAAGGCAAGTGATTTTGAAAGATTTAGGGGAAGTAATTCGGCACTTATTTTTGTTAATGAGGCTACAACTTTACACAAGCAAACTTTAGAGGAGGTCCTAAAAAGACTAAGATGCGGGCAAGAAACTATTATTTTTGATACTAATCCTGATCATCCAGAACACTATTTTAAAACCGATTATATTGATAATATAGCGACATTTAAGACATATAATTTTACAACTTATGATAATGTTCTACTTAGTAAAGGATTTATCGAAACACAAGAAAAACTCTATAAAGATATACCATCATATAAAGCAAGAGTTTTGCTAGGTGAGTGGATAGCAAGCACTGATTCAATTTTTACACAAATAAATATTACTGATGATTATGTATTTACTAGTCCAATAGCATATTTAGACCCAGCATTTAGTGTTGGAGGAGATAACACTGCATTATGTGTTATGGAGCGAGTTGATGATAAGTATTATGCTTTTGTATTTCAAGACCAACGACCAGCCAATGATCCTTATATTATGAATATGGTAAAGACCGTTATAGAAAATTTCAATGTGCATACACTGTATTTAGAGGATAGAGATAATACAAAAGGTGCTGGTGGATTGACCCGCGAATACATCTTACTAAGAAATAATATAAGCCAATATTTTAGAATTGTTCCAGTTAAGCCAAAGTCTAATAAATTTAGCAGAATAACAACGTTAATTACGCCGTTTACTTATAAGAAACTTTACATTACAAAGTACAGTAGTTCTTCTGTATTTAATGATATTTATTCGTATAAGGGGGATAGCAAAACCCATGATGATGCTCTTGATGCAATGTCTGCAGCATATTTGATGTTGTCTTTAGGATATAGAGAGCGAAGTGTTCACTTTGGCAATCAAAGATTTTTGTAA

**>CP32-4** - **phage terminase large subunit – 28947-30299**

GTGAACTTATATCAAACAAAACTTTTTACAACACTACAAAAGGAATACAAAAATAAATATGGAGTTGACATATCACAATTTGTAAAGCTAACAAATTCTTCAATTAATTTTGATAAGTTTGAAAAAGAACAGTTAACCATAAAACAAAAAAATGTTATAAAAAGTATTCAAAAAAATAATGAAAAGAAGATTATACTCAGCGGCGGCATAGCTAGCGGCAAAACGTATCTTGCATGTTATCTTTTTCTCAAAAGTTTAATTGAAAATAAAAAGTTATACTCTGGTGATATTAATAATTTTATTATTGGGAATTCACAACGCTCAGTTGAAGTTAATGTTTTGGGACAATTTGAAAAGCTATGTAAACTTCTTAAAATTCCTTATATCCCAAGACATACAAATAATTCATATATATTAATTGATTCACTTCGTATTAATCTATATGGAGGAGATAAGGCAAGTGATTTTGAAAGATTTAGAGGCAGTAATTCGGCACTTATTTTTGTTAATGAGGCTACTACTTTACACAAGCAAACTTTAGAGGAGGTCTTAAAAAGACTTAGGTGCGGACAAGAAACTATTATTTTTGATACTAATCCTGATCATCCAGAACACTATTTTAAAACCGATTATATTGATAATATAGCGACATTTAAGACATATAATTTTACAACTTATGATAATGTGCTACTTAGTAAAGGATTTATCGAAACACAAGAAAAACTCTATAAAGATATACCATCATATAAAGCAAGAGTTTTGCTAGGTGAGTGGATAGCAAGCACCGATTCAATTTTTACACAAATAAATATTACTAATGATTATGTATTTACTAGCCCGATAGCATATTTAGACCCAGCATTTAGTGTTGGAGGGGATAACACTGCATTATGTGTTATGGAGCGAGTTGATGATAAGTATTATGCTTTTGTATTTCAAGACCAACGACCAGCCAATGATCCTTATATTATGAATATGGTAAAGACCGTTATAGAAAATTTCAATGTGCATACACTGTATTTAGAGGATAGAGATAATACAAAAGGTGCTGGTGGATTGACCCGCGAATACATCTTGCTAAGAAATAATATAAGCCAATATTTTAGAATTGTTCCAGTTAAGCCAAAGTCTAATAAATTTAGCAGAATAACAACGGTAATTACGCCGTTTACTTATAAGAAACTTTACATTACAAAGTACAGTAGTTCTTCTGTATTTAATGATATTTATTCGTATAAGGGGGATAGCAAAACCCATGATGATGCTCTTGATGCAATGTCTGCAGCATATTTGATGTTGTCTTTAGGATATAGAGAGCGAAGTGTTCACTTTGGCAATCAAAGATTTTTGTAA

**>CP32-6** - **phage terminase large subunit – 28486-29838**

GTGAACTTATATCAAACAAAACTTTTTACAACACTACAAAAGGAATACAAAAATAAATATGGAGTTGATATATCACAATTTGTAAAGCTAACAAATTCTTCAATTAATTTTGATAAGTTTGAAGAAAAACAGTTAACTTTAAAACAAAAAAATGTGATAAAAAGTATTAAAAAGAATAATGAAAAAAAGATTATACTCAGCGGCGGCATAGCTAGCGGCAAAACGTATCTTGCATGCTATCTTTTTCTCAAAAGTTTAATTGAAAATAAAAAGTTATATTCTAGCGATACGAATAATTTTATTATTGGGAATTCACAACGCTCAGTTGAAGTTAATGTTTTGGGACAATTTGAAAAGCTATGTAAACTTCTTAAAATTCCTTATATTCCAAGACATACAAATAATTCATATATTCTGATTGATTCACTACGTATTAATCTATATGGTGGAGATAAGGCAAGTGATTTTGAAAGATTTAGGGGAAGTAATTCGGCACTTATTTTTGTTAATGAGGCTACAACTTTACACAAGCAAACTTTAGAGGAGGTCTTAAAAAGACTAAGATGCGGGCAAGAAACTATTATTTTTGATACTAATCCCGATCATCCAGAACACTATTTTAAAACCGATTATATTGATAATATAGCGACCTTTAAGACATATAATTTTACAACTTATGATAATGTGCTACTTAGTAAAGGATTTGTCGAAACACAAGAAAAGCTATATAAAGATATACCATCATATAAAGCAAGAGTTTTGCTAGGTGAGTGGATAGCAAGCACTGATTCAATTTTTACACAAATAAATATTACTGATGATTATGTATTTACTAGCCCGATAGCATATTTAGACCCAGCATTTAGTGTTGGCGGGGATAACACTGCATTATGTGTTATGGAGCGAGTTGATGATAAGTATTATGCTTTTGTATTTCAAGACCAAAGACCAGCTAATGATCCTTATATTATGAATATGGTAAAGACTGTTATAGAAAATTTCAATGTGCATACACTGTATTTAGAGGATAGAGATAATACAAAAGGTGCTGGTGGATTGACCCGTGAATACATCTTGCTAAGAAGTAATATAAGCCAATATTTTAGAATTGTTCCAGTTAAGCCAAAGTCTAATAAATTTAGCAGAATAACAACGTTAATTACGCCGTTTACTTACAAAAAACTTTACATTACAAAGTACAGTAGTTCTTCTGTATTTAATGATATTTATTCGTATAAGGGGGATAGCAAAACCCATGATGATGCTCTTGATGCAATGTCTGCAGCATATTTGATGTTGTCTTTAGGATATAGAGAGCGAAGTGTTCACTTTGGCAATCAAAGATTTTTGTAA

**>CP32-7** - **phage terminase large subunit – 29448-30800**

GTGAACTTATATCAAACAAAACTTTTTACAACACTACAAAAGGAATACAAAAATAAATATGGAGTTGATATATCGCAATTTATAAAGCTAACAAATTCTTCAATTAATTTTGATAAGTTTGAAGAAGAACAGCTAACTTTAAAACAAAAAAATGTGATAAAAAGCATTAAAAAGAATAATGAAAAGAAGATTATACTCAGTGGAGGTATAGCTAGCGGCAAAACGTATCTTGCATGTTATCTTTTTCTCAAAAGTTTAATTGAAAATAAAAAGCTATATTCTAGCGATACGAATAATTTCATTATAGGGAATTCACAACGTTCAGTTGAAGTTAATGTTTTGGGACAATTTGAAAAGCTATGTAAACTTCTTAAAATTCCTTATATTCCAAGACATACAAATAATTCATATATTTTGATTGATTCACTACGTATTAATCTATATGGTGGAGATAAGGCAAGTGATTTTGAAAGATTTAGGGGAAGTAATTCAGCACTTATTTTTGTGAATGAGGCTACAACTTTACACAAGCAAACTTTAGAGGAAGTCTTAAAAAGACTAAGGTGTGGGCAAGAAACTATTATTTTTGATACTAACCCCGATCATCCGGAACACTATTTTAAAACCGATTATATTGATAATATAGCAACTTTTAAGACATATAATTTTACAACTTATGATAATGTTCTACTTAGCAAAGGATTTATCGAAACTCAAGAAAAGCTATATAAAGATATACCATCATATAAAGCAAGAGTTTTGCTAGGTGAATGGATAGCAAGCACCGATTCAATTTTTACACAAATAAATATTACTAATGATTATGTATTTACTAGCCCGATAGCATATTTAGACCCAGCATTTAGTGTTGGAGGGGATAACACTGCATTATGTGTTATGGAGCGAGTTGATGATAAGTATTATGCTTTTGTATTTCAAGACCAAAGACCAGCCAATGACCCGTATATTATGAATATGGTTAAGACCGTTTTAGAGAATTTTAATGTACATACACTTTATTTAGAAGATAGAGACAATACAAAAGGTGCTGGTGGATTGACTCGTGAATACATGTTGCTAAGAAATAATATGGGTCAATATTTTAGAATTGTTCCAGTTAAGCCAAAGTCTAATAAATTTAGCAGAATAACAACGTTAATTACGCCGTTTACTTATAAGAAACTTTACATTACAAAGTACAGCAGTTCTTCTGTATTTAATGATATTTATTCGTATAAAGGAGATAACAAAACCCATGATGATGCTCTTGATGCAATATCTGCAGCATATTTGATGTTGTCTTTAGGGTATAGAGAGAGAAGTGTTCACTTTGGCAATCAAAGATTTTTGTAA

**>CP32-8** - **phage terminase large subunit – 29533-30885**

GTGAACTTATATCAAACAAAACTTTTTACAACACTACAAAAGGAATACAAAAATAAATATGGAGTTGATATATCACAATTTGTAAAGCTAACAAATTCTTCAATTAATTTTGATAAGTTTGAAGAAGAACAGTTAACTTTAAAACAAAAAAATGTGATAAAAAGCATTAAAAAGAATAATGAAAAGAAGATTATACTCAGCGGAGGCATAGCTAGTGGCAAAACGTATCTTGCATGTTATCTTTTTCTAAAAAGTTTAATTGAAAATAAAAAGTTATACTCTAGTGATACTAATAATTTCATTATAGGGAATTCACAACGTTCAGTTGAAGTTAATGTTTTGGGGCAATTTGAAAAGCTATGTAAACTTCTTAAAATTCCTTATATTCCAAGACATACAAATAATTCATATATTCTGATTGATTCACTACGTATTAATCTATATGGAGGAGATAAGGCAAGTGATTTTGAAAGATTTAGGGGAAGTAATTCGGCACTTATTTTTGTTAATGAGGCTACAACTTTACACAAGCAAACTTTAGAGGAAGTCTTAAAAAGACTAAGATGCGGGCAAGAAACTATTATTTTTGATACTAATCCTGATCATCCAGAACACTATTTTAAAACCGATTATATTGATAATATAGCGACCTTTAAGACATATAATTTACAACTTATGATAATGTGCTACTTAGTAAAGGATTTGTCGAAACACAAGAAAAGCTATATAAAGATATACCATCATATAAAGCAAGAGTTTTGTTAGGTGAGTGGATAGCAAGCACTGATTCAATTTTTACACAAATAAATATTACTGATGATTATGTATTTACTAGCCCGATAGCATATTTAGACCCAGCATTTAGTGTTGGAGGGGATAACACTGCATTATGTGTTATGGAGCGAGTTGATGATAAGTATTATGCTTTTGTATTTCAAGACCAAAGACCAGCTAATGATCCTTATATTATGAATATGGTAAAGACTGTTATAGAAAATTTCAATGTGCATACACTGTATTTAGAGGATAGAGATAATACAAAAGGTGCTGGTGGATTGACCCGTGAATACATCTTGCTAAGAAGTAATATAAGCCAATATTTTAGAATTGTTCCAGTTAAGCCAAAGTCTAATAAATTTAGCAGAATAACAACGTTAATTACGCCGTTTACTTACAAAAAACTTTATATTACAAAGTACAGTAGTTCTTCCGTATTTAATGATATTTATTCGTATAAGGGGGATAATAAAACCCATGATGACGCTCTTGATGCAATATCTGCAGCATATTTGATGTTGTCTTTAGGATATAGAGAGCGAAGTGTTCACT

TTGGCAATCAAAGATTTTTGTAA

**>CP32-9** - **phage terminase large subunit – 29299-30651**

GTGAACTTATATCAAACAAAACTTTTTACAACACTACAAAAGGAATACAAAAATAAATATGGAGTTGACATATCACAATTTGTAAAGCTAGCAAATTCTTCAATTAATTTTGATAAGTTTGAAGAAAAACAGTTAACTTTAAAACAAAAAAATGTGATAAAAAGTATTAAAAAGAATAATGAAAAAAAGATTATACTCAGCGGAGGCATAGCTAGTGGCAAAACGTATCTTGCATGTTATCTTTTTCTAAAAAGTTTAATTGAAAATAAAAAGTTATACTCTAGTGATACTAATAATTTCATTATTGGGAATTCACAACGCTCAGTTGAAGTTAATGTTTTAGGACAATTTGAAAAGCTATGTAAACTTCTTAAAATTCCTTATATCCCAAGACATACAAATAATTCATATATTCTGATTGATTCACTACGTATTAATCTATATGGTGGAGATAAGGCAAGTGATTTTGAAAGATTTAGGGGAAGTAATTCAGCACTTATTTTTGTTAATGAGGCTACAACTTTACACAAGCAAACTTTAGAGGAGGTCTTAAAAAGACTAAGATGCGGGCAAGAAACTATTATTTTTGATACTAATCCTGATCATCCAGAACACTATTTTAAAACCGATTATATTGATAATATAGCGACATTTAAGATATATAATTTTACAACTTATGATAATGTGCTACTTAGTAAAGGATTTATCGAAACACAAGAAAAACTCTATAAAGATATACCATCATATAAAGCAAGAGTTTTGCTAGGTGAGTGGATAGCAAGCACTGATTCAATTTTTACACAAATAAATATTACTGATGATTATATATTTACTAGCCCGATAGCATATTTAGACCCAGCATTTAGTGTTGGAGGGGATAACACTGCATTATGTGTTATGGAGCGAGTTGATGATAAGTATTATGCTTTTGTATTTCAAGACCAACGACCAGCCAATGATCCTTATATTATGAATATGGTAAAGACCGTTATAGAAAATTTCAATGTGCATACACTGTATTTAGAGGATAGAGATAATACAAAAGGTGCTGGTGGATTGACCCGCGAATACATCTTGCTAAGAAATAATATAAGCCAATATTTTAGAATTGTTCCAGTTAAGCCAAAGTCTAATAAATTTAGCAGAATAACAATGTTAATTACGCCGTTTACTTACAAAAAACTTTATATTACAAAGTACAGTAGTTCTTCTGTATTTAATGATATTTATTCGTATAAGGGGGATAGCAAAACCCATGATGATGCTCTTGATGCAATATCTGCAGCATATTTGATGTTGTCTTTAGGATATAGAGAGCGAAGTGTTCACTTTGGCAATCAAAGATTTTTGTAA

**>LP56** - **phage terminase large subunit - 32455-33807**

GTGAACTTATATCAAACAAAACTTTTTACAATACTACAAAAGGAATACAAAAATAAATATGGAGTTGATATATCACAATTTGTAAAGCTAACAAATTCTTCAATTAATTTTGATAAGTTTGAAGAAGAGCAGCTAACTTTAAAACAAAAAAATGTGATAAAAAGCATTAAAAAGAATAATGAAAAAAAGATTATACTCAGCGGCGGCATAGCTAGCGGCAAAACGTATCTTGCATGTTATCTTTTTATAAAAAGTTTAATTGAAAATAAAAAGCTATATTCTAGCGATACGAATAATTTCATTATAGGGAATTCACAACGCTCAGTTGAAGTTAATGTTTTGGGGCAATTTGAAAAGCTATGCAAACTGCTTAAAATTCCTTATATTCCAAGACATACAAATAATTCATATATTCTGATTGATTCACTGCGAATTAATCTACATGGTGGAGATAAGGCAAGTGATTTTGAAAGATTTAGGGGAAGTAATTCAGCACTTATTTTTGTTAATGAGGCTACAACTTTACACAAGCAAACTTTAGAGGAAGTCTTAAAAAGATTAAGATGCGGGCAAGAAACTATTATTTTTGATACTAATCCTGATCATCCAGAACACTATTTTAAAACCGATTATATTGATAATATAGCGACCTTTAAGACATATAATTTTACAACTTATGATAATGTTCTACTTAGTAAAGGATTTATCGAAACACAAGAAAAGCTCTATAAAGATATACCATCATATAAAGCAAGAGTTTTGCTAGGGGAGTGGATAGCAAGCACCGATTCAATTTTTACACAAATAAATATTACTAATGATTATGTATTTACTAGCCCGATAGCATATTTAGACCCAGCATTTAGTGTTGGCGGGGATAACACTGCATTATGTGTTATGGAGCGGGTTGATGATAAGTATTATGCTTTTGTATTTCAAGACCAACGACCAGCCAATGACCCGTATATTATGAATATGGTTAAGACCGTTTTAGAAAATTTTAATGTACATACATTTTATTTAGAAGATAGAGACAATACAAAAGGTGCTGGTGGATTGACCCGCGAATACATCTTGCTAAGAAATAATATGGGTCAATATTTTAGAATTGTTCCAGTTAAGCCAAAGTCTAATAAATTTAGCAGAATAACAGCGTTAATTACGCCGTTTATTTATAAGAAACTGTACATTACGAAGTACAGCAGTTCTTCTGTATTTAATGATATTTATTCGTATAAAGGAGATAACAAAACCCATGATGATGCTCTTGATGCAATATCTGCAGCATATTTGATGTTGTCTTTAGGGTATAGAGAGAGAAGTGTTCACTTTGGCAATCAAAGATTTTTGTAA

**>LP54** - **pbsx family phage terminase – NC_001857.2 (18725..20077) (this one is only distantly similar to the above eight terminase sequences, therefore not possible to include this one in the primers and probe design)**

TTGAAATTCTTAAGATCATTAGCGTTTCTCAATCTTCAGAAAAAGTTTAAAAATAAGTTTAATATTAACATTTTGGATTATATTAAGCCAAAACCAACCAAAATTTGTTTTAAAGATTTTGAAAATAAATACTTAACTGCTAAGCAAAAGGAAGTTCTTTTTGACATAGAAAGTAACAATTATTCAAAAGTAATATTTAGCGGTGGGATTGCAAGTGGTAAGACGTTTTTAGCTTCATATTTGCTTGTTAAAAAGCTTATTGAAAACAAATCTTTTTATGAGCAAGATACCAATAATTTTATTATAGGCAACTCAATTGGTTTGTTGATGACAAATACTGTAAAGCAAATAGAGAAAATTTGCAGCTTGCTGGGAATTGATTACGAGAAAAAGAAAAGCGGGCAGTCTTTTTGTAAGATAGCAGGTCTTAAGCTTAATATTTACGGGGGTAAAAACAGAGATGCTTTTTCCAAGATTCGTGGGGGCAATAGTGCAATAATTTATGTAAATGAAGCAACAGTAATTCACAGAGAAACTTTACTTGAAGTAATAAAAAGGCTTAGAAAGGGTAAAGAAATTATTATTTTTGATACAAATCCGGAAAGCCCTGCGCATTATTTTAAAACCGATTATATTGAAAATACAGATGTTTTTAAGACATATAATTTTACAACTTATGACAATCCTTTAAATTCAGCAGATTTTATTCAAACTCAGGAAAAACTTTACAGGCGTTTTCCCGCCTATAGGGCTCGTGTGCTTTACGGGGAGTGGATTTTAAATGAATCTACGCTATTTAATGAGATGATTTTCAATCAAGATTATGAATTTAAAAGCCCAATAATGTACATTGATCCTGCATTCTCAGTGGGTGGAGACAATACAGCTATTTGTGTTTTAGAGCGCACTTTTGAGAAGTTTTATGCATATATTTATCAAGACCAAAAACCAGTAAGTGATAGTTTAATGCTTGCTTCCATTCAAGTTTTAATAGAAAATTTCAATGTAAATACCGTTTACATTGAGGAGAGAGACAGTACCAAGGGGGATGGAATTTTAACTAAAACAATTCTTTTCTTGAGAAACAAAAGCAGTCACTATTTTAAAGTTGCACCAATAAAACCTTTAAGCAATAAATTTAAAAGAATATGCGCGCTTATTCCTTTGTTTGAAAGCCGCAAAATAGAATTTTTAAAAATAATAAGTAAAAATGTAATATCTGATATTTACAGCTACAAAGGAGATGGCAAAACAAAAGACGATGCGCTTGATTCTCTTGCAAATGCGTATCTTCTTTTAACGCTAAATTATAAAGAAAAATTATTTCATTTCGGCAGGTTTAAATATTTATAA

**>LP28-2** – **Hypothetical protein – NC_001852.1 (16494..17684, complement) (this one is not annotated as termiase gene)**

ATGAGACTAAGGCGACTTCCAGTATATGTTGATGCCTACAAAGAAAAGCCCAATGCTGAAATTTTCATATACTACTCAAGTAGGGGAACTGGTAAAACCTACGACATTGCAACTGTTAATTTAGAAAGAAAATTTAGTGCTGATGGCGGAGATACCCTTGCAATTAGAAAAAAGAAAAACAAAACAACACAATCAATACACAAAGAAATTTTAGAACTTTTAAGCATATACAACTTAAGAAAATTTTTCAATATAAGCAAAGCAAAAATTGAAAGTAAGAGTTTGATTTTTGGGAAAAAACGTGCTTTTGTTTTTGAAGGGGGGCATGATACAAGAGATTTAAAATCTTATGCGCATTTTAAGGACTTATGGCTGGAAGAAGCCAATCAGTTTAGCGCTGATGATATAGAAATGCTTGTCCCTACAATGAGAGAACAAGGCGGCAGAATCTATATGTCAAGCAATCCGGTGCCTAAATCACATTGGCTATACAAAAGGTACTTATCAAATCAAGACAATCCTGCTGTGTGTATAATCAAAAGCACTTACCGTGACAACCCATTTTTAAATGGTGGAGACGTACAAGCTTGGCTTGAAAAACAAAGACTTGCATATCATGGCAATGACATTGGTTTTAGAATTGAGGTTTTAGGAGAAGAGTTTGATTTTGGTACAGCAAGGCTAATTAAAAAATTTAATGTGTGTGGTCCCGAAATTCTTTCCAGAGCTAATGGAAGCTATTATACAGGAATACACGTTAAAGGCAACAGAATTTGTTTTTTAGAAATTCTTGTTGGAAGAATTTCCTATCTTCCAGTTGTAATTATTACAAACGCATGTAGTAAAGTTTTACTATCAAAAACTGATTACCAATCCGAAATTAATAAATTTAAAGGGGTTTTTGTATTGCCAACAGCAAGAGAAGAACTCAAATATGTATTTTCTCGTTTTGGTAGAGGTACTTTGCTTGCAAAAAAACGAAACTTGTATTCACTCTCAGACTATTTGATTCCGTCTAATCTTAATGTGGTAAACAAACCCGAAACCACTGATGTAATTTCAGAGTTCAACGAAACTGAATATTATTATGATGAATCTAGTGCAGAAGATAGCGAAGTTACAAATTTTGTTATGCAAAAAGATTTGGTATACATCCCGGCATTTCTCAATGCCATATCGGTTTTTAGCTAA

**NT Alignment of phage terminase large subunit from all CP32 Plasmids & LP56.**

CP32-1 GTGAACTTATATCAAACAAAACTTTTTACAACACTACAAAAGGAATACAAAAATAAATAT 60

CP32-8 GTGAACTTATATCAAACAAAACTTTTTACAACACTACAAAAGGAATACAAAAATAAATAT 60

CP32-6 GTGAACTTATATCAAACAAAACTTTTTACAACACTACAAAAGGAATACAAAAATAAATAT 60

CP32-4 GTGAACTTATATCAAACAAAACTTTTTACAACACTACAAAAGGAATACAAAAATAAATAT 60

CP32-9 GTGAACTTATATCAAACAAAACTTTTTACAACACTACAAAAGGAATACAAAAATAAATAT 60

CP32-3 GTGAACTTATATCAAACAAAACTTTTTACAACACTACAAAAGGAATACAAAAATAAATAT 60

CP32-7 GTGAACTTATATCAAACAAAACTTTTTACAACACTACAAAAGGAATACAAAAATAAATAT 60

LP56 GTGAACTTATATCAAACAAAACTTTTTACAATACTACAAAAGGAATACAAAAATAAATAT 60

******************************* ****************************

CP32-1 GGAGTTGATATATCACAATTTGTAAAGCTAACAAATTCTTCAATTAATTTTGATAAGTTT 120

CP32-8 GGAGTTGATATATCACAATTTGTAAAGCTAACAAATTCTTCAATTAATTTTGATAAGTTT 120

CP32-6 GGAGTTGATATATCACAATTTGTAAAGCTAACAAATTCTTCAATTAATTTTGATAAGTTT 120

CP32-4 GGAGTTGACATATCACAATTTGTAAAGCTAACAAATTCTTCAATTAATTTTGATAAGTTT 120

CP32-9 GGAGTTGACATATCACAATTTGTAAAGCTAGCAAATTCTTCAATTAATTTTGATAAGTTT 120

CP32-3 GGAGTTGATATATCACAATTTGTAAAGCTAACAAATTCTTCAATTAATTTTGATAAGTTT 120

CP32-7 GGAGTTGATATATCGCAATTTATAAAGCTAACAAATTCTTCAATTAATTTTGATAAGTTT 120

LP56 GGAGTTGATATATCACAATTTGTAAAGCTAACAAATTCTTCAATTAATTTTGATAAGTTT 120

******** ***** ****** ******** *****************************

CP32-1 GAAGAAGAACAGTTAACTTTAAAACAAAAAAATGTGATAAAAAGCATTAAAAAGAATAAT 180

CP32-8 GAAGAAGAACAGTTAACTTTAAAACAAAAAAATGTGATAAAAAGCATTAAAAAGAATAAT 180

CP32-6 GAAGAAAAACAGTTAACTTTAAAACAAAAAAATGTGATAAAAAGTATTAAAAAGAATAAT 180

CP32-4 GAAAAAGAACAGTTAACCATAAAACAAAAAAATGTTATAAAAAGTATTCAAAAAAATAAT 180

CP32-9 GAAGAAAAACAGTTAACTTTAAAACAAAAAAATGTGATAAAAAGTATTAAAAAGAATAAT 180

CP32-3 GAAGAAAAACAGTTAACTTTAAAACAAAAAAATGTGATAAAAAGTATTAAAAAGAATAAT 180

CP32-7 GAAGAAGAACAGCTAACTTTAAAACAAAAAAATGTGATAAAAAGCATTAAAAAGAATAAT 180

LP56 GAAGAAGAGCAGCTAACTTTAAAACAAAAAAATGTGATAAAAAGCATTAAAAAGAATAAT 180

*** ** * *** **** **************** ******** *** **** ******

CP32-1 GAAAAGAAGATTATACTCAGCGGAGGCATAGCTAGTGGCAAAACGTATCTTGCATGTTAT 240

CP32-8 GAAAAGAAGATTATACTCAGCGGAGGCATAGCTAGTGGCAAAACGTATCTTGCATGTTAT 240

CP32-6 GAAAAAAAGATTATACTCAGCGGCGGCATAGCTAGCGGCAAAACGTATCTTGCATGCTAT 240

CP32-4 GAAAAGAAGATTATACTCAGCGGCGGCATAGCTAGCGGCAAAACGTATCTTGCATGTTAT 240

CP32-9 GAAAAAAAGATTATACTCAGCGGAGGCATAGCTAGTGGCAAAACGTATCTTGCATGTTAT 240

CP32-3 GAAAAAAAGATTATATTCAGCGGCGGCATAGCTAGCGGCAAAACGTATCTTGCATGCTAT 240

CP32-7 GAAAAGAAGATTATACTCAGTGGAGGTATAGCTAGCGGCAAAACGTATCTTGCATGTTAT 240

LP56 GAAAAAAAGATTATACTCAGCGGCGGCATAGCTAGCGGCAAAACGTATCTTGCATGTTAT 240

***** ********* **** ** ** ******** ******************** ***

CP32-1 CTTTTTCTAAAAAGTTTAATTGAAATTAAAAAGTTATACTCTAGTGATACTAATAATTTC 300

CP32-8 CTTTTTCTAAAAAGTTTAATTGAAAATAAAAAGTTATACTCTAGTGATACTAATAATTTC 300

CP32-6 CTTTTTCTCAAAAGTTTAATTGAAAATAAAAAGTTATATTCTAGCGATACGAATAATTTT 300

CP32-4 CTTTTTCTCAAAAGTTTAATTGAAAATAAAAAGTTATACTCTGGTGATATTAATAATTTT 300

CP32-9 CTTTTTCTAAAAAGTTTAATTGAAAATAAAAAGTTATACTCTAGTGATACTAATAATTTC 300

CP32-3 CTTTTTCTCAAAAGTTTAATTGAAAATAAAAAGTTATATTCTAGCGATACGAATAATTTT 300

CP32-7 CTTTTTCTCAAAAGTTTAATTGAAAATAAAAAGCTATATTCTAGCGATACGAATAATTTC 300

LP56 CTTTTTATAAAAAGTTTAATTGAAAATAAAAAGCTATATTCTAGCGATACGAATAATTTC 300

****** * **************** ******* **** *** * **** ********

CP32-1 ATTATAGGGAATTCACAACGTTCAGTTGAAGTTAATGTTTTGGGGCAATTTGAAAAGCTA 360

CP32-8 ATTATAGGGAATTCACAACGTTCAGTTGAAGTTAATGTTTTGGGGCAATTTGAAAAGCTA 360

CP32-6 ATTATTGGGAATTCACAACGCTCAGTTGAAGTTAATGTTTTGGGACAATTTGAAAAGCTA 360

CP32-4 ATTATTGGGAATTCACAACGCTCAGTTGAAGTTAATGTTTTGGGACAATTTGAAAAGCTA 360

CP32-9 ATTATTGGGAATTCACAACGCTCAGTTGAAGTTAATGTTTTAGGACAATTTGAAAAGCTA 360

CP32-3 ATTATTGGGAATTCACAACGCTCAGTTGAAGTTAATGTTTTGGGACAATTTGAAAAGCTA 360

CP32-7 ATTATAGGGAATTCACAACGTTCAGTTGAAGTTAATGTTTTGGGACAATTTGAAAAGCTA 360

LP56 ATTATAGGGAATTCACAACGCTCAGTTGAAGTTAATGTTTTGGGGCAATTTGAAAAGCTA 360

***** ************** ******************** ** ***************

CP32-1 TGTAAACTTCTTAAAATTCCTTATATTCCAAGACATACAAATAATTCATATATTCTGATT 420

CP32-8 TGTAAACTTCTTAAAATTCCTTATATTCCAAGACATACAAATAATTCATATATTCTGATT 420

CP32-6 TGTAAACTTCTTAAAATTCCTTATATTCCAAGACATACAAATAATTCATATATTCTGATT 420

CP32-4 TGTAAACTTCTTAAAATTCCTTATATCCCAAGACATACAAATAATTCATATATATTAATT 420

CP32-9 TGTAAACTTCTTAAAATTCCTTATATCCCAAGACATACAAATAATTCATATATTCTGATT 420

CP32-3 TGTAAACTTCTTAAAATTCCTTATATTCCAAGACATACAAATAATTCATATATTCTGATT 420

CP32-7 TGTAAACTTCTTAAAATTCCTTATATTCCAAGACATACAAATAATTCATATATTTTGATT 420

LP56 TGCAAACTGCTTAAAATTCCTTATATTCCAAGACATACAAATAATTCATATATTCTGATT 420

** ***** ***************** ************************** * ***

CP32-1 GATTCACTACGTATTAATCTATATGGAGGAGATAAGGCAAGTGATTTTGAAAGATTTAGG 480

CP32-8 GATTCACTACGTATTAATCTATATGGAGGAGATAAGGCAAGTGATTTTGAAAGATTTAGG 480

CP32-6 GATTCACTACGTATTAATCTATATGGTGGAGATAAGGCAAGTGATTTTGAAAGATTTAGG 480

CP32-4 GATTCACTTCGTATTAATCTATATGGAGGAGATAAGGCAAGTGATTTTGAAAGATTTAGA 480

CP32-9 GATTCACTACGTATTAATCTATATGGTGGAGATAAGGCAAGTGATTTTGAAAGATTTAGG 480

CP32-3 GATTCACTACGTATTAATCTATATGGTGGAGATAAGGCAAGTGATTTTGAAAGATTTAGG 480

CP32-7 GATTCACTACGTATTAATCTATATGGTGGAGATAAGGCAAGTGATTTTGAAAGATTTAGG 480

LP56 GATTCACTGCGAATTAATCTACATGGTGGAGATAAGGCAAGTGATTTTGAAAGATTTAGG 480

******** ** ********* **** ********************************

CP32-1 GGAAGTAATTCGGCACTTATTTTTGTTAATGAGGCTACAACTTTACACAAGCAAACTTTA 540

CP32-8 GGAAGTAATTCGGCACTTATTTTTGTTAATGAGGCTACAACTTTACACAAGCAAACTTTA 540

CP32-6 GGAAGTAATTCGGCACTTATTTTTGTTAATGAGGCTACAACTTTACACAAGCAAACTTTA 540

CP32-4 GGCAGTAATTCGGCACTTATTTTTGTTAATGAGGCTACTACTTTACACAAGCAAACTTTA 540

CP32-9 GGAAGTAATTCAGCACTTATTTTTGTTAATGAGGCTACAACTTTACACAAGCAAACTTTA 540

CP32-3 GGAAGTAATTCGGCACTTATTTTTGTTAATGAGGCTACAACTTTACACAAGCAAACTTTA 540

CP32-7 GGAAGTAATTCAGCACTTATTTTTGTGAATGAGGCTACAACTTTACACAAGCAAACTTTA 540

LP56 GGAAGTAATTCAGCACTTATTTTTGTTAATGAGGCTACAACTTTACACAAGCAAACTTTA 540

** ******** ************** *********** *********************

CP32-1 GAGGAAGTCTTAAAAAGACTAAGATGCGGGCAAGAAACTATTATTTTTGATACTAATCCT 600

CP32-8 GAGGAAGTCTTAAAAAGACTAAGATGCGGGCAAGAAACTATTATTTTTGATACTAATCCT 600

CP32-6 GAGGAGGTCTTAAAAAGACTAAGATGCGGGCAAGAAACTATTATTTTTGATACTAATCCC 600

CP32-4 GAGGAGGTCTTAAAAAGACTTAGGTGCGGACAAGAAACTATTATTTTTGATACTAATCCT 600

CP32-9 GAGGAGGTCTTAAAAAGACTAAGATGCGGGCAAGAAACTATTATTTTTGATACTAATCCT 600

CP32-3 GAGGAGGTCCTAAAAAGACTAAGATGCGGGCAAGAAACTATTATTTTTGATACTAATCCT 600

CP32-7 GAGGAAGTCTTAAAAAGACTAAGGTGTGGGCAAGAAACTATTATTTTTGATACTAACCCC 600

LP56 GAGGAAGTCTTAAAAAGATTAAGATGCGGGCAAGAAACTATTATTTTTGATACTAATCCT 600

***** *** ******** * ** ** ** ************************** **

CP32-1 GATCATCCAGAACACTATTTTAAAACCGATTATATTGATAATATAGCGACCTTTAAGACA 660

CP32-8 GATCATCCAGAACACTATTTTAAAACCGATTATATTGATAATATAGCGACCTTTAAGACA 660

CP32-6 GATCATCCAGAACACTATTTTAAAACCGATTATATTGATAATATAGCGACCTTTAAGACA 660

CP32-4 GATCATCCAGAACACTATTTTAAAACCGATTATATTGATAATATAGCGACATTTAAGACA 660

CP32-9 GATCATCCAGAACACTATTTTAAAACCGATTATATTGATAATATAGCGACATTTAAGATA 660

CP32-3 GATCATCCAGAACACTATTTTAAAACCGATTATATTGATAATATAGCGACATTTAAGACA 660

CP32-7 GATCATCCGGAACACTATTTTAAAACCGATTATATTGATAATATAGCAACTTTTAAGACA 660

LP56 GATCATCCAGAACACTATTTTAAAACCGATTATATTGATAATATAGCGACCTTTAAGACA 660

******** ************************************** ** ******* *

CP32-1 TATAAGTTTACAACTTATGATAATGTGCTACTTAGTAAAGGATTTGTCGAAACACAAGAA 720

CP32-8 TATAA-TTTACAACTTATGATAATGTGCTACTTAGTAAAGGATTTGTCGAAACACAAGAA 719

CP32-6 TATAATTTTACAACTTATGATAATGTGCTACTTAGTAAAGGATTTGTCGAAACACAAGAA 720

CP32-4 TATAATTTTACAACTTATGATAATGTGCTACTTAGTAAAGGATTTATCGAAACACAAGAA 720

CP32-9 TATAATTTTACAACTTATGATAATGTGCTACTTAGTAAAGGATTTATCGAAACACAAGAA 720

CP32-3 TATAATTTTACAACTTATGATAATGTTCTACTTAGTAAAGGATTTATCGAAACACAAGAA 720

CP32-7 TATAATTTTACAACTTATGATAATGTTCTACTTAGCAAAGGATTTATCGAAACTCAAGAA 720

LP56 TATAATTTTACAACTTATGATAATGTTCTACTTAGTAAAGGATTTATCGAAACACAAGAA 720

***** ******************** ******** ********* ******* ******

CP32-1 AAGCTATATAAAGATATACCATCATATAAAGCAAGAGTTTTGTTAGGTGAGTGGATAGCA 780

CP32-8 AAGCTATATAAAGATATACCATCATATAAAGCAAGAGTTTTGTTAGGTGAGTGGATAGCA 779

CP32-6 AAGCTATATAAAGATATACCATCATATAAAGCAAGAGTTTTGCTAGGTGAGTGGATAGCA 780

CP32-4 AAACTCTATAAAGATATACCATCATATAAAGCAAGAGTTTTGCTAGGTGAGTGGATAGCA 780

CP32-9 AAACTCTATAAAGATATACCATCATATAAAGCAAGAGTTTTGCTAGGTGAGTGGATAGCA 780

CP32-3 AAACTCTATAAAGATATACCATCATATAAAGCAAGAGTTTTGCTAGGTGAGTGGATAGCA 780

CP32-7 AAGCTATATAAAGATATACCATCATATAAAGCAAGAGTTTTGCTAGGTGAATGGATAGCA 780

LP56 AAGCTCTATAAAGATATACCATCATATAAAGCAAGAGTTTTGCTAGGGGAGTGGATAGCA 780

** ** ************************************ **** ** *********

CP32-1 AGCACTGATTCAATTTTTACACAAATAAATATTACTGATGATTATGTATTTACTAGCCCG 840

CP32-8 AGCACTGATTCAATTTTTACACAAATAAATATTACTGATGATTATGTATTTACTAGCCCG 839

CP32-6 AGCACTGATTCAATTTTTACACAAATAAATATTACTGATGATTATGTATTTACTAGCCCG 840

CP32-4 AGCACCGATTCAATTTTTACACAAATAAATATTACTAATGATTATGTATTTACTAGCCCG 840

CP32-9 AGCACTGATTCAATTTTTACACAAATAAATATTACTGATGATTATATATTTACTAGCCCG 840

CP32-3 AGCACTGATTCAATTTTTACACAAATAAATATTACTGATGATTATGTATTTACTAGTCCA 840

CP32-7 AGCACCGATTCAATTTTTACACAAATAAATATTACTAATGATTATGTATTTACTAGCCCG 840

LP56 AGCACCGATTCAATTTTTACACAAATAAATATTACTAATGATTATGTATTTACTAGCCCG 840

***** ****************************** ******** ********** **

CP32-1 ATAGCATATTTAGACCCAGCATTTAGTGTTGGCGGGGATAACACTGCATTATGTGTTATG 900

CP32-8 ATAGCATATTTAGACCCAGCATTTAGTGTTGGAGGGGATAACACTGCATTATGTGTTATG 899

CP32-6 ATAGCATATTTAGACCCAGCATTTAGTGTTGGCGGGGATAACACTGCATTATGTGTTATG 900

CP32-4 ATAGCATATTTAGACCCAGCATTTAGTGTTGGAGGGGATAACACTGCATTATGTGTTATG 900

CP32-9 ATAGCATATTTAGACCCAGCATTTAGTGTTGGAGGGGATAACACTGCATTATGTGTTATG 900

CP32-3 ATAGCATATTTAGACCCAGCATTTAGTGTTGGAGGAGATAACACTGCATTATGTGTTATG 900

CP32-7 ATAGCATATTTAGACCCAGCATTTAGTGTTGGAGGGGATAACACTGCATTATGTGTTATG 900

LP56 ATAGCATATTTAGACCCAGCATTTAGTGTTGGCGGGGATAACACTGCATTATGTGTTATG 900

******************************** ** ************************

CP32-1 GAGCGAGTTGATGATAAGTATTATGCTTTTGTATTTCAAGACCAAAGACCAGCTAATGAT 960

CP32-8 GAGCGAGTTGATGATAAGTATTATGCTTTTGTATTTCAAGACCAAAGACCAGCTAATGAT 959

CP32-6 GAGCGAGTTGATGATAAGTATTATGCTTTTGTATTTCAAGACCAAAGACCAGCTAATGAT 960

CP32-4 GAGCGAGTTGATGATAAGTATTATGCTTTTGTATTTCAAGACCAACGACCAGCCAATGAT 960

CP32-9 GAGCGAGTTGATGATAAGTATTATGCTTTTGTATTTCAAGACCAACGACCAGCCAATGAT 960

CP32-3 GAGCGAGTTGATGATAAGTATTATGCTTTTGTATTTCAAGACCAACGACCAGCCAATGAT 960

CP32-7 GAGCGAGTTGATGATAAGTATTATGCTTTTGTATTTCAAGACCAAAGACCAGCCAATGAC 960

LP56 GAGCGGGTTGATGATAAGTATTATGCTTTTGTATTTCAAGACCAACGACCAGCCAATGAC 960

***** *************************************** ******* *****

CP32-1 CCTTATATTATGAATATGGTAAAGACTGTTATAGAAAATTTCAATGTGCATACACTGTAT 1020

CP32-8 CCTTATATTATGAATATGGTAAAGACTGTTATAGAAAATTTCAATGTGCATACACTGTAT 1019

CP32-6 CCTTATATTATGAATATGGTAAAGACTGTTATAGAAAATTTCAATGTGCATACACTGTAT 1020

CP32-4 CCTTATATTATGAATATGGTAAAGACCGTTATAGAAAATTTCAATGTGCATACACTGTAT 1020

CP32-9 CCTTATATTATGAATATGGTAAAGACCGTTATAGAAAATTTCAATGTGCATACACTGTAT 1020

CP32-3 CCTTATATTATGAATATGGTAAAGACCGTTATAGAAAATTTCAATGTGCATACACTGTAT 1020

CP32-7 CCGTATATTATGAATATGGTTAAGACCGTTTTAGAGAATTTTAATGTACATACACTTTAT 1020

LP56 CCGTATATTATGAATATGGTTAAGACCGTTTTAGAAAATTTTAATGTACATACATTTTAT 1020

** ***************** ***** *** **** ***** ***** ****** * ***

CP32-1 TTAGAGGATAGAGATAATACAAAAGGTGCTGGTGGATTGACCCGTGAATACATCTTGCTA 1080

CP32-8 TTAGAGGATAGAGATAATACAAAAGGTGCTGGTGGATTGACCCGTGAATACATCTTGCTA 1079

CP32-6 TTAGAGGATAGAGATAATACAAAAGGTGCTGGTGGATTGACCCGTGAATACATCTTGCTA 1080

CP32-4 TTAGAGGATAGAGATAATACAAAAGGTGCTGGTGGATTGACCCGCGAATACATCTTGCTA 1080

CP32-9 TTAGAGGATAGAGATAATACAAAAGGTGCTGGTGGATTGACCCGCGAATACATCTTGCTA 1080

CP32-3 TTAGAGGATAGAGATAATACAAAAGGTGCTGGTGGATTGACCCGCGAATACATCTTACTA 1080

CP32-7 TTAGAAGATAGAGACAATACAAAAGGTGCTGGTGGATTGACTCGTGAATACATGTTGCTA 1080

LP56 TTAGAAGATAGAGACAATACAAAAGGTGCTGGTGGATTGACCCGCGAATACATCTTGCTA 1080

***** ******** ************************** ** ******** ** ***

CP32-1 AGAAGTAATATAAGCCAATATTTTAGAATTGTTCCAGTTAAGCCAAAGTCTAATAAATTT 1140

CP32-8 AGAAGTAATATAAGCCAATATTTTAGAATTGTTCCAGTTAAGCCAAAGTCTAATAAATTT 1139

CP32-6 AGAAGTAATATAAGCCAATATTTTAGAATTGTTCCAGTTAAGCCAAAGTCTAATAAATTT 1140

CP32-4 AGAAATAATATAAGCCAATATTTTAGAATTGTTCCAGTTAAGCCAAAGTCTAATAAATTT 1140

CP32-9 AGAAATAATATAAGCCAATATTTTAGAATTGTTCCAGTTAAGCCAAAGTCTAATAAATTT 1140

CP32-3 AGAAATAATATAAGCCAATATTTTAGAATTGTTCCAGTTAAGCCAAAGTCTAATAAATTT 1140

CP32-7 AGAAATAATATGGGTCAATATTTTAGAATTGTTCCAGTTAAGCCAAAGTCTAATAAATTT 1140

LP56 AGAAATAATATGGGTCAATATTTTAGAATTGTTCCAGTTAAGCCAAAGTCTAATAAATTT 1140

**** ****** * *********************************************

CP32-1 AGCAGAATAACAACGTTAATTACGCCGTTTACTTACAAAAAACTTTATATTACAAAGTAC 1200

CP32-8 AGCAGAATAACAACGTTAATTACGCCGTTTACTTACAAAAAACTTTATATTACAAAGTAC 1199

CP32-6 AGCAGAATAACAACGTTAATTACGCCGTTTACTTACAAAAAACTTTACATTACAAAGTAC 1200

CP32-4 AGCAGAATAACAACGGTAATTACGCCGTTTACTTATAAGAAACTTTACATTACAAAGTAC 1200

CP32-9 AGCAGAATAACAATGTTAATTACGCCGTTTACTTACAAAAAACTTTATATTACAAAGTAC 1200

CP32-3 AGCAGAATAACAACGTTAATTACGCCGTTTACTTATAAGAAACTTTACATTACAAAGTAC 1200

CP32-7 AGCAGAATAACAACGTTAATTACGCCGTTTACTTATAAGAAACTTTACATTACAAAGTAC 1200

LP56 AGCAGAATAACAGCGTTAATTACGCCGTTTATTTATAAGAAACTGTACATTACGAAGTAC 1200

************ * *************** *** ** ***** ** ***** ******

CP32-1 AGTAGTTCTTCCGTATTTAATGATATTTATTCGTATAAGGGGGATAATAAAACCCATGAT 1260

CP32-8 AGTAGTTCTTCCGTATTTAATGATATTTATTCGTATAAGGGGGATAATAAAACCCATGAT 1259

CP32-6 AGTAGTTCTTCTGTATTTAATGATATTTATTCGTATAAGGGGGATAGCAAAACCCATGAT 1260

CP32-4 AGTAGTTCTTCTGTATTTAATGATATTTATTCGTATAAGGGGGATAGCAAAACCCATGAT 1260

CP32-9 AGTAGTTCTTCTGTATTTAATGATATTTATTCGTATAAGGGGGATAGCAAAACCCATGAT 1260

CP32-3 AGTAGTTCTTCTGTATTTAATGATATTTATTCGTATAAGGGGGATAGCAAAACCCATGAT 1260

CP32-7 AGCAGTTCTTCTGTATTTAATGATATTTATTCGTATAAAGGAGATAACAAAACCCATGAT 1260

LP56 AGCAGTTCTTCTGTATTTAATGATATTTATTCGTATAAAGGAGATAACAAAACCCATGAT 1260

** ******** ************************** ** **** ************

CP32-1 GACGCTCTTGATGCAATATCTGCAGCATATTTGATGTTGTCTTTAGGATATAGAGAGCGA 1320

CP32-8 GACGCTCTTGATGCAATATCTGCAGCATATTTGATGTTGTCTTTAGGATATAGAGAGCGA 1319

CP32-6 GATGCTCTTGATGCAATGTCTGCAGCATATTTGATGTTGTCTTTAGGATATAGAGAGCGA 1320

CP32-4 GATGCTCTTGATGCAATGTCTGCAGCATATTTGATGTTGTCTTTAGGATATAGAGAGCGA 1320

CP32-9 GATGCTCTTGATGCAATATCTGCAGCATATTTGATGTTGTCTTTAGGATATAGAGAGCGA 1320

CP32-3 GATGCTCTTGATGCAATGTCTGCAGCATATTTGATGTTGTCTTTAGGATATAGAGAGCGA 1320

CP32-7 GATGCTCTTGATGCAATATCTGCAGCATATTTGATGTTGTCTTTAGGGTATAGAGAGAGA 1320

LP56 GATGCTCTTGATGCAATATCTGCAGCATATTTGATGTTGTCTTTAGGGTATAGAGAGAGA 1320

** ************** ***************************** ********* **

CP32-1 AGTGTTCACTTTGGCAATCAAAGATTTTTGTAA 1353

CP32-8 AGTGTTCACTTTGGCAATCAAAGATTTTTGTAA 1352

CP32-6 AGTGTTCACTTTGGCAATCAAAGATTTTTGTAA 1353

CP32-4 AGTGTTCACTTTGGCAATCAAAGATTTTTGTAA 1353

CP32-9 AGTGTTCACTTTGGCAATCAAAGATTTTTGTAA 1353

CP32-3 AGTGTTCACTTTGGCAATCAAAGATTTTTGTAA 1353

CP32-7 AGTGTTCACTTTGGCAATCAAAGATTTTTGTAA 1353

LP56 AGTGTTCACTTTGGCAATCAAAGATTTTTGTAA 1353

*********************************

**Supplementary information 2:**  A phylogenetic tree constructed based on the *terL* nucleotide sequence showing that the LD, RF *Borrelia* species and *B. miyamotoi* are separated to three clades. Molecular Evolutionary Genetics Analysis (MEGA) package version 7 with maximum likelihood method was used. Scale bar represents the units of the number of base substitutions per site. Support for clades was estimated using a bootstrap analysis in MEGA with 3,000 replicates, values are indicated at the nodes (only values greater than 75 were displayed). The *terL* sequence from phage lambda was used as an outgroup to root the tree.

**Supplementary information 3: the *terL* nucleotide sequence from 13 cp32 plasmids present within *Borrelia burgdorferi* species**

13 versions of the *terL* genes are discovered from 13 cp32 plasmids. The alignment shows that they are similar to each other, which allows primers (yellow and green) and probe (purple) to be designed in the conserved regions of nine out of 13 terminase genes (except cp32-2, cp32-3, cp32-6 and cp32-13) with the help of PrimerQuest® Tool (IDT).

>cp32-1 AE001575.1:29398-30750 Borrelia burgdorferi B31 plasmid cp32-1, complete sequence

GTGAACTTATATCAAACAAAACTTTTTACAACACTACAAAAGGAATACAAAAATAAATATGGAGTTGATATATCACAATT

TGTAAAGCTAACAAATTCTTCAATTAATTTTGATAAGTTTGAAGAAGAACAGTTAACTTTAAAACAAAAAAATGTGATAA

AAAGCATTAAAAAGAATAATGAAAAGAAGATTATACTCAGCGGAGGCATAGCTAGTGGCAAAACGTATCTTGCATGTTAT

CTTTTTCTAAAAAGTTTAATTGAAATTAAAAAGTTATACTCTAGTGATACTAATAATTTCATTATAGGGAATTCACAACG

TTCAGTTGAAGTTAATGTTTTGGGGCAATTTGAAAAGCTATGTAAACTTCTTAAAATTCCTTATATTCCAAGACATACAA

ATAATTCATATATTCTGATTGATTCACTACGTATTAATCTATATGGAGGAGATAAGGCAAGTGATTTTGAAAGATTTAGG

GGAAGTAATTCGGCACTTATTTTTGTTAATGAGGCTACAACTTTACACAAGCAAACTTTAGAGGAAGTCTTAAAAAGACT

AAGATGCGGGCAAGAAACTATTATTTTTGATACTAATCCTGATCATCCAGAACACTATTTTAAAACCGATTATATTGATA

ATATAGCGACCTTTAAGACATATAAGTTTACAACTTATGATAATGTGCTACTTAGTAAAGGATTTGTCGAAACACAAGAA

AAGCTATATAAAGATATACCATCATATAAAGCAAGAGTTTTGTTAGGTGAGTGGATAGCAAGCACTGATTCAATTTTTAC

ACAAATAAATATTACTGATGATTATGTATTTACTAGCCCGATAGCATATTTAGACCCAGCATTTAGTGTTGGCGGGGATA

ACACTGCATTATGTGTTATGGAGCGAGTTGATGATAAGTATTATGCTTTTGTATTTCAAGACCAAAGACCAGCTAATGAT

CCTTATATTATGAATATGGTAAAGACTGTTATAGAAAATTTCAATGTGCATACACTGTATTTAGAGGATAGAGATAATAC

AAAAGGTGCTGGTGGATTGACCCGTGAATACATCTTGCTAAGAAGTAATATAAGCCAATATTTTAGAATTGTTCCAGTTA

AGCCAAAGTCTAATAAATTTAGCAGAATAACAACGTTAATTACGCCGTTTACTTACAAAAAACTTTATATTACAAAGTAC

AGTAGTTCTTCCGTATTTAATGATATTTATTCGTATAAGGGGGATAATAAAACCCATGATGACGCTCTTGATGCAATATC

TGCAGCATATTTGATGTTGTCTTTAGGATATAGAGAGCGAAGTGTTCACTTTGGCAATCAAAGATTTTTGTAA

>cp32-5 CP019849.1:29322-30674 Borreliella burgdorferi plasmid cp32-5, complete sequence

GTGAACTTATATCAAACAAAACTTTTTACAACACTACAAAAGGAATACAAAAATAAATATGGAGTTGATATATCACAATT

TGTAAAGCTAACAAATTCTTCAATTAATTTTGATAAGTTTGAAGAAGAACAGTTAACTTTAAAACAAAAAAATGTGATAA

AAAGCATTAAAAAGAATAATGAAAAGAAGATTATACTCAGCGGAGGCATAGCTAGTGGCAAAACGTATCTTGCATGTTAT

CTTTTTCTAAAAAGTTTAATTGAAAATAAAAAGTTATACTCTAGTGATACTAATAATTTCATTATAGGGAATTCACAACG

TTCAGTTGAAGTTAATGTTTTGGGGCAATTTGAAAAGCTATGTAAACTTCTTAAAATTCCTTATATTCCAAGACATACAA

ATAATTCATATATTCTGATTGATTCACTACGTATTAATCTATATGGAGGAGATAAGGCAAGTGATTTTGAAAGATTTAGG

GGAAGTAATTCGGCACTTATTTTTGTTAATGAGGCTACAACTTTACACAAGCAAACTTTAGAGGAAGTCTTAAAAAGACT

AAGATGCGGGCAAGAAACTATTATTTTTGATACTAATCCTGATCATCCAGAACACTATTTTAAAACCGATTATATTGATA

ATATAGCGACCTTTAAGACATATAAGTTTACAACTTATGATAATGTGCTACTTAGTAAAGGATTTGTCGAAACACAAGAA

AAGCTATATAAAGATATACCATCATATAAAGCAAGAGTTTTGTTAGGTGAGTGGATAGCAAGCACTGATTCAATTTTTAC

ACAAATAAATATTACTGATGATTATGTATTTACTAGCCCGATAGCATATTTAGACCCAGCATTTAGTGTTGGCGGGGATA

ACACTGCATTATGTGTTATGGAGCGAGTTGATGATAAGTATTATGCTTTTGTATTTCAAGACCAAAGACCAGCTAATGAT

CCTTATATTATGAATATGGTAAAGACTGTTATAGAAAATTTCAATGTGCATACACTGTATTTAGAGGATAGAGATAATAC

AAAAGGTGCTGGTGGATTGACCCGTGAATACATCTTGCTAAGAAGTAATATAAGCCAATATTTTAGAATTGTTCCAGTTA

AGCCAAAGTCTAATAAATTTAGCAGAATAACAACGTTAATTACGCCGTTTACTTACAAAAAACTTTATATTACAAAGTAC

AGTAGTTCTTCCGTATTTAATGATATTTATTCGTATAAGGGGGATAATAAAACCCATGATGACGCTCTTGATGCAATATC

TGCAGCATATTTGATGTTGTCTTTAGGATATAGAGAGCGAAGTGTTCACTTTGGCAATCAAAGATTTTTGTAA

>cp32-8 AE001580.1:29533-30885 Borrelia burgdorferi B31 plasmid cp32-8, complete sequence

GTGAACTTATATCAAACAAAACTTTTTACAACACTACAAAAGGAATACAAAAATAAATATGGAGTTGATATATCACAATT

TGTAAAGCTAACAAATTCTTCAATTAATTTTGATAAGTTTGAAGAAGAACAGTTAACTTTAAAACAAAAAAATGTGATAA

AAAGCATTAAAAAGAATAATGAAAAGAAGATTATACTCAGCGGAGGCATAGCTAGTGGCAAAACGTATCTTGCATGTTAT

CTTTTTCTAAAAAGTTTAATTGAAAATAAAAAGTTATACTCTAGTGATACTAATAATTTCATTATAGGGAATTCACAACG

TTCAGTTGAAGTTAATGTTTTGGGGCAATTTGAAAAGCTATGTAAACTTCTTAAAATTCCTTATATTCCAAGACATACAA

ATAATTCATATATTCTGATTGATTCACTACGTATTAATCTATATGGAGGAGATAAGGCAAGTGATTTTGAAAGATTTAGG

GGAAGTAATTCGGCACTTATTTTTGTTAATGAGGCTACAACTTTACACAAGCAAACTTTAGAGGAAGTCTTAAAAAGACT

AAGATGCGGGCAAGAAACTATTATTTTTGATACTAATCCTGATCATCCAGAACACTATTTTAAAACCGATTATATTGATA

ATATAGCGACCTTTAAGACATATAAGTTTACAACTTATGATAATGTGCTACTTAGTAAAGGATTTGTCGAAACACAAGAA

AAGCTATATAAAGATATACCATCATATAAAGCAAGAGTTTTGTTAGGTGAGTGGATAGCAAGCACTGATTCAATTTTTAC

ACAAATAAATATTACTGATGATTATGTATTTACTAGCCCGATAGCATATTTAGACCCAGCATTTAGTGTTGGAGGGGATA

ACACTGCATTATGTGTTATGGAGCGAGTTGATGATAAGTATTATGCTTTTGTATTTCAAGACCAAAGACCAGCTAATGAT

CCTTATATTATGAATATGGTAAAGACTGTTATAGAAAATTTCAATGTGCATACACTGTATTTAGAGGATAGAGATAATAC

AAAAGGTGCTGGTGGATTGACCCGTGAATACATCTTGCTAAGAAGTAATATAAGCCAATATTTTAGAATTGTTCCAGTTA

AGCCAAAGTCTAATAAATTTAGCAGAATAACAACGTTAATTACGCCGTTTACTTACAAAAAACTTTATATTACAAAGTAC

AGTAGTTCTTCCGTATTTAATGATATTTATTCGTATAAGGGGGATAATAAAACCCATGATGACGCTCTTGATGCAATATC

TGCAGCATATTTGATGTTGTCTTTAGGATATAGAGAGCGAAGTGTTCACTTTGGCAATCAAAGATTTTTGTAA

>cp32-3 CP002269.1:28910-30262 Borrelia burgdorferi 297 plasmid 297_cp32-3, complete sequence

GTGAACTTATATCAAACAAAACTTTTTACAACACTACAAAAGGAATATAAAAATAAATATGGAGTTGACATATCACAATT

TGTAAAGCTAACAAATTCTTCAATTAATTTTGATAAGTTTGAAGAAAAACAGTTAACTTTAAAACAAAAAAATGTGATAA

AAAGCATTAAAAAGAATAATGAAAAGAAGATTATACTCAGCGGAGGCATAGCTAGTGGCAAAACGTATCTTGCATGTTAT

CTTTTTCTAAAAAGTTTAATTGAAAATAAAAAGTTATACTCTAGTGATACTAATAATTTTATTATTGGGAATTCACAACG

CTCAGTTGAAGTTAATGTTTTGGGACAATTTGAAAAGCTATGTAAACTTCTTAAAATTCCTTATATCCCAAGACATACAA

ATAATTCATATATTCTGATTGATTCACTACGTATTAATCTATATGGTGGAGATAAGGCAAGTGATTTTGAAAGATTTAGG

GGAAGTAATTCGGCACTTATTTTTGTTAATGAGGCTACAACTTTACACAAGCAAACTTTAGAGGAGGTCTTAAAAAGACT

AAGATGCGGGCAAGAAACTATTATTTTTGATACTAATCCTGATCATCCAGAACACTATTTTAAAACCGATTATATTGATA

ATATAGCGACCTTTAAGACATATAATTTTACAACTTATGATAATGTGCTACTTAGTAAAGGATTTGTCGAAACACAAGAA

AAGCTATATAAAGATATACCATCATATAAAGCAAGAGTTTTGTTAGGTGAGTGGATAGCAAGCACCGATTCAATTTTTAC

ACAAATAAATATTACTGATGATTATGTATTTACTAGCCCGATAGCATATTTAGACCCAGCATTTAGTGTTGGCGGGGATA

ACACTGCATTATGTGTTATGGAGCGAGTTGATGATAAGTATTATGCTTTTGTATTTCAAGACCAAAGACCAGCTAATGAT

CCTTATATTATGAATATGGTAAAGACTGTTATAGAAAATTTCAATGTGCATACACTGTATTTAGAGGATAGAGATAATAC

AAAAGGTGCTGGTGGATTGACCCGTGAATACATCTTGCTAAGAAGTAATATAAGCCAATATTTTAGAATTGTTCCAGTTA

AGCCAAAGTCTAATAAATTTAGCAGAATAACAACGTTAATTACGCCGTTTACTTACAAAAAACTTTATATTACAAAGTAC

AGTAGTTCTTCCGTATTTAATGATATTTATTCGTATAAGGGGGATAATAAAACCCATGATGATGCTCTTGATGCAATGTC

TGCAGCATATTTGATGTTGTCTTTAGGATATAGAGAGCGAAGTGTTCACTTTGGCAATCAAAGATTTTTGTAA

>cp32-4 CP001208.1:29612-30964 Borrelia burgdorferi ZS7 plasmid ZS7_cp32-4, complete sequence

GTGAACTTATATCAAACAAAACTTTTTACAACACTACAAAAGGAATATAAAAATAAATATGGAGTTGACATATCACAATT

TGTAAAGCTAACAAATTCTTCAATTAATTTTGATAAGTTTGAAGAAAAACAGTTAACTTTAAAACAAAAAAATGTGATAA

AAAGCATTCAAAAGAATAATGAAAAGAAGATTATACTCAGCGGAGGCATAGCTAGTGGCAAAACGTATCTTGCATGTTAT

CTTTTTCTAAAAAGTTTAATTGAAAATAAAAAGTTATACTCTAGTGATACTAATAATTTTATTATTGGGAATTCACAACG

CTCAGTTGAAGTTAATGTTTTGGGACAATTTGAAAAGCTATGTAAACTTCTTAAAATTCCTTATATCCCAAGACATACAA

ATAATTCATATATTCTGATTGATTCACTACGTATTAATCTATATGGTGGAGATAAGGCAAGTGATTTTGAAAGATTTAGG

GGAAGTAATTCGGCACTTATTTTTGTTAATGAGGCTACAACTTTACACAAGCAAACTTTAGAGGAAGTCTTAAAAAGACT

AAGATGCGGGCAAGAAACTATTATTTTTGATACTAATCCTGATCATCCAGAACACTATTTTAAAACCGATTATATTGATA

ATATAGCGACCTTTAAGACATATAAGTTTACAACTTATGATAATGTGCTACTTAGTAAAGGATTTGTCGAAACACAAGAA

AAGCTATATAAAGATATACCATCATATAAAGCAAGAGTTTTGTTAGGTGAGTGGATAGCAAGCACTGATTCAATTTTTAC

ACAAATAAATATTACTGATGATTATGTATTTACTAGCCCGATAGCATATTTAGACCCAGCATTTAGTGTTGGCGGGGATA

ACACTGCATTATGTGTTATGGAGCGAGTTGATGATAAGTATTATGCTTTTGTATTTCAAGACCAAAGACCAGCTAATGAT

CCTTATATTATGAATATGGTAAAGACTGTTATAGAAAATTTCAATGTGCATACACTGTATTTAGAGGATAGAGATAATAC

AAAAGGTGCTGGTGGATTGACCCGTGAATACATCTTGCTAAGAAGTAATATAAGCCAATATTTTAGAATTGTTCCAGTTA

AGCCAAAGTCTAATAAATTTAGCAGAATAACAACGTTAATTACGCCGTTTACTTACAAAAAACTTTATATTACAAAGTAC

AGTAGTTCTTCTGTATTTAATGATATTTATTCGTATAAGGGGGATAGCAAAACCCATGATGATGCTCTTGATGCAATGTC

TGCAGCATATTTGATGTTGTCTTTAGGATATAGAGAGCGAAGTGTTCACTTTGGCAATCAAAGATTTTTGTAA

>cp32-7 CP017209.1:28702-30054 Borreliella burgdorferi strain B331 plasmid B331_cp32_7

GTGAACTTATATCAAACAAAACTTTTTACAACACTACAAAAGGAATACAAAAATAAATATGGAGTTGACATATCACAATT

TGTAAAGCTAACAAATTCTTCAATTAATTTTGATAAGTTTGAAGAAAAACAGTTAACTTTAAAACAAAAAAATGTGATAA

AAAGCATTCAAAAGAATAATGAAAAGAAGATTATACTCAGCGGAGGCATAGCTAGTGGCAAAACGTATCTTGCATGTTAT

CTTTTTCTAAAAAGTTTAATTGAAAATAAAAAGTTATACTCTAGTGATACTAATAATTTTATTATTGGGAATTCACAACG

CTCAGTTGAAGTTAATGTTTTGGGACAATTTGAAAAGCTATGTAAACTTCTTAAAATTCCTTATATCCCAAGACATACAA

ATAATTCATATATTCTGATTGATTCACTACGTATTAATCTATATGGTGGAGATAAGGCAAGTGATTTTGAAAGATTTAGG

GGAAGTAATTCAGCACTTATTTTTGTTAATGAGGCTACAACTTTACACAAGCAAACTTTAGGGGAGGTCTTAAAAAGACT

AAGATGCGGGCAAGAAACTATTATTTTTGATACCAATCCTGATCATCCAGAACACTATTTTAAAACCGATTATATTGATA

ATATAGCGACCTTTAAGACATATAATTTTACAACTTATGATAATGTGCTACTTAGTAAAGGATTTGTCGAAACACAAGAA

AAGCTATATAAAGATATACCATCATATAAAGCAAGAGTTTTGCTAGGTGAGTGGATAGCAAGCACTGATTCAATTTTTAC

ACAAATAAATATTACTGATGATTATGTATTTACTAGCCCAATAGCATATTTAGACCCAGCATTTAGTGTTGGAGGAGATA

ACACTGCATTATGTGTTATGGAGCGAGTTGATGATAAGTATTATGCTTTTGTATTTCAAGACCAACGACCGGCCAATGAT

CCTTATATTATGAATATGGTAAAGACTGTTATAGAAAATTTCAATGTGCATACACTGTATTTAGAGGATAGAGATAATAC

AAAAGGTGCTGGTGGATTGACCCGTGAATACATCTTGCTAAGAAGTAATATAAGCCAATATTTTAGAATTGTTCCAGTTA

AGCCAAAGTCTAATAAATTTAGCAGAATAACAACGTTAATTACGCCGTTTACTTACAAAAAACTTTATATTACAAAGTAC

AGTAGTTCTTCTGTATTTAATGATATTTATTCGTATAAGGGGGATAGCAAAACCCATGATGATGCTCTTGATGCAATGTC

TGCAGCATATTTGATGTTGTCTTTAGGATATAGAGAGCGAAGTGTTCACTTTGGCAATCAAAGATTTTTGTAA

>cp32-11 CP001426.1:28908-30260 Borrelia burgdorferi 64b plasmid 64b_cp32-11, complete sequence

GTGAACTTATATCAAACAAAACTTTTTACAACACTACAAAAGGAATACAAAAATAAATATGGAGTTGATATATCACAATT

TGTAAAGCTAACAAATTCTTCAATTAATTTTGATAAGTTTGAAGAAGAACAGTTAACTTTAAAACAAAAAAATGTGATAA

AAAGCATTAAAAAGAATAATGAAAAGAAGATTATACTCAGTGGAGGTATAGCTAGTGGCAAAACGTATCTTGCATGTTAT

CTTTTTCTAAAAAGTTTAATTAAAAATAAAAAGTTATACTCTAGTGATACTAATAATTTCATTATAGGGAATTCACAACG

TTCAGTTGAAGTTAATGTTTTGGGGCAATTTGAAAAGCTATGTAAACTTCTTAAAATTCCTTATATTCCAAGACATACAA

ATAATTCATATATTCTGATTGATTCACTGCGAATTAATCTATATGGTGGAGATAAGGCAAGTGATTTTGAAAGATTTAGG

GGAAGTAATTCAGCACTTATTTTTGTTAATGAGGCTACAACTTTACACAAGCAAACTTTAGAGGAGGTCTTAAAAAGACT

AAGATGCGGGCAAGAAACTATTATTTTTGATACTAATCCCGATCATCCAGAACACTATTTTAAAACCGATTATATTGATA

ATATAGCGACCTTTAAGACATATAATTTTACAACTTATGATAATGTGCTACTTAGTAAAGGATTTGTCGAAACACAAGAA

AAGCTATATAAAGATATACCATCATATAAAGCAAGAGTTTTGCTAGGTGAGTGGATAGCAAGCACTGATTCAATTTTTAC

ACAAATAAATATTACTGATGATTATGTATTTACTAGCCCGATAGCATATTTAGACCCAGCATTTAGTGTTGGCGGGGATA

ACACTGCATTATGTGTTATGGAGCGAGTTGATGATAAGTATTATGCTTTTGTATTTCAAGACCAAAGACCAGCTAATGAT

CCTTATATTATGAATATGGTAAAGACCGTTATAGAAAATTTCAATGTGCATACACTGTATTTAGAGGATAGAGATAATAC

AAAAGGTGCTGGTGGATTGACCCGTGAATACATCTTGCTAAGAAGTAATATAAGCCAATATTTTAGAATTGTTCCAGTTA

AGCCAAAGTCTAATAAATTTAGCAGAATAACAACGTTAATTACGCCGTTTACTTACAAAAAACTTTATATTACAAAGTAC

AGTAGTTCTTCTGTATTTAATGATATTTATTCGTATAAGGGGGATAGCAAAACCCATGATGATGCTCTTGATGCAATGTC

TGCAGCATATTTGATGTTGTCTTTAGGATATAGAGAGCGAAGTGTTCACTTTGGCAATCAAAGATTTTTGTAA

>cp32-6 CP001427.1:29403-30755 Borrelia burgdorferi 64b plasmid 64b_cp32-6, complete sequence

GTGAACTTATATCAAACAAAACTTTTTACAACACTACAAAAGGAATATAAAAATAAATATGGAGTTGACATATCACAATT

TGTAAAGCTAACAAATTCTTCAATTAATTTTGATAAGTTTGAAGAAAAACAGTTAACTTTAAAACAAAAAAATGTGATAA

AAAGCATTCAAAAGAATAATGAAAAGAAGATTATACTCAGCGGAGGCATAGCTAGTGGCAAAACGTATCTTGCATGTTAT

CTTTTTCTAAAAAGTTTAATTGAAAATAAAAAGTTATACTCTAGTGATACTAATAATTTCATTATAGGGAATTCACAACG

TTCAGTTGAAGTTAATGTTTTGGGACAATTTGAAAAGCTATGTAAACTTCTTAAAATTCCTTATATCCCAAGACATACAA

ATAATTCATATATTCTGATTGATTCACTACGTATTAATCTATATGGTGGAGATAAGGCAAGTGATTTTGAAAGATTTAGG

GGAAGTAATTCGGCACTTATTTTTGTTAATGAGGCTACAACTTTACACAAGCAAACTTTAGAGGAAGTCTTAAAAAGACT

AAGATGCGGGCAAGAAACTATTATTTTTGATACTAATCCTGATCATCCAGAACACTATTTTAAAACCGATTATATTGATA

ATATAGCGACCTTTAAGACATATAATTTTACAACTTATGATAATGTGCTACTTAGTAAAGGATTTGTCGAAACACAAGAA

AAGCTATATAAAGATATACCATCATATAAAGCAAGAGTTTTGTTAGGTGAGTGGATAGCAAGCACTGATTCAATTTTTAC

ACAAATAAATATTACTGATGATTATGTATTTACTAGCCCAATAGCATATTTAGACCCAGCATTTAGTGTTGGAGGAGATA

ACACTGCATTATGTGTTATGGAGCGAGTTGATGATAAGTATTATGCTTTTGTATTTCAAGACCAACGACCAGCCAATGAT

CCTTATATTATGAATATCGTAAAGACTGTTATAGAAAATTTCAATGTGCATACACTGTATTTAGAGGATAGAGATAATAC

AAAAGGTGCTGGTGGATTGACCCGTGAATACATCTTACTAAGAAGTAATATAAGCCAATATTTTAGAATTGTTCCAGTTA

AGCCAAAGTCTAATAAATTTAGCAGAATAACAACGTTAATTACGCCGTTTACTTACAAAAAACTTTACATTACAAAGTAC

AGTAGTTCTTCTGTATTTAATGATATTTATTCGTATAAGGGGGATAATAAAACCCATGATGATGCTCTTGATGCAATGTC

TGCAGCATATTTGATGTTGTCTTTAGGATATAGAGAGCGAAGTGTTCACTTTGGCAATCAAAGATTTTTGTAA

>cp32-2 CP019919.1:29893-31245 Borreliella burgdorferi strain PAbe plasmid p_cp32-2, complete sequence

GTGAACTTATATCAAACAAAACTTTTTACAACACTACAAAAGGAATACAAAAATAAATATGGAGTTGATATATCACAATT

TGTAAAGCTAACAAATTCTTCAATTAATTTTGATAAGTTTGAAGAAGAACAGTTAACTTTAAAACAGAAAAATGTGATAA

AAAGCATTGAAAAGAATAATGAAAAGAAGATTATACTCAGTGGAGGTATAGCTAGCGGCAAAACGTATCTTGCATGTTAT

CTTTTTCTAAAAAGTTTAATTGAAAATAAAAAGCTATATTCTAGCGATACGAATAATTTCATTATAGGGAATTCACAACG

TTCAGTTGAAGTTAATGTTTTGGGACAATTTGAAAAGCTATGTAAACTTCTTAAAATTCCTTATATTCCAAGACATACAA

ATAATTCATATATTCTGATTGATTCACTGCGAATTAATCTATATGGTGGAGATAAGGCAAGTGTTTTTGAAAGATTTAGG

GGAAGTAATTCAGCACTTATTTTTGTGAATGAGGCTACAACTTTACACAAGCAAACTTTAGAGGAGGTCTTAAAAAGACT

AAGATGCGGGCAAGAAACTATTATTTTTGATACTAATCCTGATCATCCAGAACACTATTTTAAAACCGATTATATTGATA

ATATAGCGACCTTTAAGACATATAATTTCACAACTTATGATAATGTGCTACTTAGTAAAGGATTTGTCGAAACACAAGAA

AAGCTATATAAAGATATACCATCATATAAAGCAAGAGTTTTGCTAGGTGAATGGATAGCAAGCACCGATTCAATTTTTAC

ACAAATAAATATTACTGATGATTATGTATTTACTAGCCCGATAGCATATTTAGACCCAGCATTTAGTGTTGGAGGGGATA

ACACTGCATTATGTGTTATGGAGCGAATTGATGATAAGTATTATGCTTTTGTATTTCAAGACCAACGACCAGCCAATGAT

CCTTATATTATGAATATGGTAAAGACCGTTATAGAAAATTTCAATGTGCATACACTGTATTTAGAGGATAGAGATAATAC

AAAAGGTGCTGGTGGATTGACCCGTGAATACATCTTGCTAAGAAATAATACGAGTCAATATTTTAGAATTGTTCCAGTTA

AGCCAAAGTCTAATAAATTTAGCAGAATAACAACGTTAATTACGCCGTTTACTTATAAGAAACTTTACATTACAAAGTAC

AGTAGTTCTTCTGTATTTAATGATATTTATTCGTATAAGGGGGATAACAAAACCCATGATGATGCTCTTGATGCAATGTC

TGCAGCATATTTGATGTTGTCTTTAGGATATAGAGAGCGAAGTGTTCACTTTGGCAATCAAAGATTTTTGTAA

>cp32-12 CP001565.1:30081-31433 Borrelia burgdorferi Bol26 plasmid Bol26_cp32-12, complete sequence

GTGAACTTATATCAAACAAAACTTTTTACAACACTACAAAAGGAATACAAAAATAAATATGGAGTTGACATATCACAATT

TGTAAAGCTAACAAATTCTTCAATTAATTTTGATAAGTTTGAAGAAAAACAGTTAACTTTAAAACAAAAAAATGTGATAA

AAAGCATTAAAAAGAATAATGAAAAAAAGATTATACTCAGCGGCGGCATAGCTAGCGGCAAAACGTATCTTGCCTGTTAT

CTTTTTCTAAAAAGTTTAATTGCAAATAAGAATTTATATTCTAGTGATACGAATAATTTCATTATTGGCAATTCGCAGCG

CTCGGTTGAAGTTAATGTTTTGGGGCAATTTGAAAAGCTATGCAAACGGCTTAAAATTCCTTATATTCCAAGACATACAA

ATAATTCATATATTCTGATTGATTCACTACGTATTAATCTATATGGTGGAGATAAGGCAAGTGATTTTGAAAGATTTAGG

GGAAGTAATTCAGCGCTTATTTTTGTTAATGAGGCTACAACTTTACACAGGCAAACTTTAGAGGAAGTCTTAAAAAGACT

AAGGTGTGGGCAAGAAACTATTATTTTTGATACTAACCCCGATCATCCGGAACACTATTTTAAAACCGATTATATTGATA

ATATAGCAACTTTTAAGACATATAATTTTACAACTTATGATAATGTTCTACTTAGTAAAGGATTTATCGAAACACAAGAA

AAACTCTATAAAGATATACCATCATATAAAGCAAGAGTTTTGCTAGGTGAGTGGATAGCAAGCACTGATTCAATTTTTAC

ACAAATAAATATTACTGATGATTATGTATTTACTAGCCCGATAGCATATTTAGACCCAGCATTTAGTGTTGGCGGGGATA

ACACTGCATTATGTGTTATGGAGCGAGTTGATGATAAGTATTATGCTTTTGTATTTCAAGACCAACGACCAGCTAATGAT

CCTTATATTATGAATATGGTAAAGACCGTTATAGAAAATTTCAATGTGCATACACTGTATTTAGAGGATAGAGATAATAC

AAAAGGTGCTGGTGGATTGACCCGTGAATACATCTTGCTAAGAAATAATATAAGCCAATATTTTAGAATTGTTCCAGTTA

AGCCAAAGTCTAATAAATTTAGCAGAATAACAACGTTAATTACGCCGTTTACTTATAAGAAACTTTACATTACAAAGTAC

AGTAGTTCTTCTGTATTTAATGATATTTATTCGTATAAGGGGGATAACAAAACCCATGATGATGCTCTTGATGCAATGTC

TGCAGCATATTTGATGTTGTCTTTAGGATATAGAGAGCGAAGTGTTCACTTTGGCAACCAAAGATTTTTGTAA

>cp32-10 CP001449.1:28404-29756 Borrelia burgdorferi WI91-23 plasmid WI91-23_cp32-10, complete sequence

GTGAACTTATATCAAACAAAACTTTTTACAACACTACAAAAGGAATACAAAAATAAATATGGAGTTGATATATCACAATT

TGTAAAGCTAACAAATTCTTCAATTAATTTTGATAAGTTTGAAGAAGAACAGTTAACTTTAAAACAAAAAAATGTGATAA

AAAGCATTAAAAAGAATAATGAAAAAAAGATTATACTCAGCGGCGGCATAGCTAGCGGCAAAACGTATCTTGCATGTTAT

CTTTTTCTAAAAAGTTTAATTAAAAATAAAAAGTTATATTCTAGCGATACGAATAATTTTATTATTGGGAATTCACAACG

CTCAGTTGAAGTTAATGTTTTGGGACAATTTGAAAAGCTATGTAAACTTCTTAAAATTCCTTATATTCCAAGACATACAA

ATAATTCATATATTCTTATTGATTCACTGAGAATTAATCTATATGGAGGAGATAAGGCAAGTGATTTTGAAAGATTTAGG

GGAAGTAATTCGGCACTTATTTTTGTTAATGAGGCTACAACTTTACACAAGCAAACTTTAGAGGAGGTCTTAAAAAGACT

AAGATGCGGGCAAGAAACTATTATTTTTGATACTAATCCCGATCATCCAGAACACTATTTTAAAACCGATTATATTGATA

ATATAGCGACATTTAAGACATATAATTTTACAACTTATGATAATGTGCTACTTAGTAAAGGATTTGTCGAAACACAAGAA

AAGCTATATAAAGATATACCATCATATAAAGCAAGAGTTTTGCTAGGTGAGTGGATAGCAAGCACTGATTCAATTTTTAC

ACAAATAAATATTACTGATGATTATGTATTTACTAGCCCGATAGCATATTTAGACCCAGCATTTAGTGTTGGCGGGGATA

ACACTGCATTATGTGTTATGGAGCGAGTTGATGATAAGTATTATGCTTTTGTATTTCAAGACCAAAGACCAGCTAATGAT

CCTTATATTATGAATATGGTAAAGACTGTTATAGAAAATTTCAATGTGCATACACTGTATTTAGAGGATAGAGATAATAC

AAAAGGTGCTGGTGGATTGACCCGTGAATACATCTTGCTAAGAAGTAATATAAGCCAATATTTTAGAATTGTTCCAGTTA

AGCCAAAGTCTAATAAATTTAGCAGAATAACAACGTTAATTACGCCGTTTACTTACAAAAAACTTTATATTACAAAGTAC

AGTAGTTCTTCTGTATTTAATGATATTTATTCGTATAAGGGGGATAGCAAAACCCATGATGATGCTCTTGATGCAATGTC

TGCAGCATATTTGATGTTGTCTTTAGGATATAGAGAGCGAAGTGTTCACTTTGGCAATCAAAGATTTTTGTAA

>cp32-9 CP001211.1:29115-30467 Borrelia burgdorferi ZS7 plasmid ZS7_cp32-9, complete sequence

GTGAACTTATATCAAACAAAACTTTTTACAACACTACAAAAGGAATACAAAAATAAATATGGAGTTGACATATCACAATT

TGTAAAGCTAGCAAATTCTTCAATTAATTTTGATAAGTTTGAAGAAAAACAGTTAACTTTAAAACAAAAAAATGTGATAA

AAAGTATTAAAAAGAATAATGAAAAAAAGATTATACTCAGCGGAGGCATAGCTAGTGGCAAAACGTATCTTGCATGTTAT

CTTTTTCTAAAAAGTTTAATTGAAAATAAAAAGTTATACTCTAGTGATACTAATAATTTCATTATTGGGAATTCACAACG

CTCAGTTGAAGTTAATGTTTTAGGACAATTTGAAAAGCTATGTAAACTTCTTAAAATTCCTTATATCCCAAGACATACAA

ATAATTCATATATTCTGATTGATTCACTACGTATTAATCTATATGGTGGAGATAAGGCAAGTGATTTTGAAAGATTTAGG

GGAAGTAATTCAGCACTTATTTTTGTGAATGAGGCTACAACTTTACACAAGCAAACTTTAGAGGAGGTCTTAAAAAGACT

AAGATGCGGGCAAGAAACTATTATTTTTGATACTAATCCTGATCATCCAGAACACTATTTTAAAACCGATTATATTGATA

ATATAGCGACATTTAAGACATATAATTTTACAACTTATGATAATGTGCTACTTAGTAAAGGATTTATCGAAACACAAGAA

AAACTCTATAAAGATATACCATCATATAAAGCAAGAGTTTTGCTAGGTGAGTGGATAGCAAGCACTGATTCAATTTTTAC

ACAAATAAATATTACTGATGATTATATATTTACTAGCCCGATAGCATATTTAGACCCAGCATTTAGTGTTGGAGGGGATA

ACACTGCATTATGTGTTATGGAGCGAGTTGATGATAAGTATTATGCTTTTGTATTTCAAGACCAACGACCAGCCAATGAT

CCTTATATTATGAATATGGTAAAGACCGTTATAGAAAATTTCAATGTGCATACACTGTATTTAGAGGATAGAGATAATAC

AAAAGGTGCTGGTGGATTGACCCGCGAATACATCTTGCTAAGAAATAATATAAGCCAATATTTTAGAATTGTTCCAGTTA

AGCCAAAGTCTAATAAATTTAGCAGAATAACAACGTTAATTACGCCGTTTACTTACAAAAAACTTTATATTACAAAGTAC

AGTAGTTCTTCTGTATTTAATGATATTTATTCGTATAAGGGGGATAGCAAAACCCATGATGATGCTCTTGATGCAATATC

TGCAGCATATTTGATGTTGTCTTTAGGATATAGAGAGCGAAGTGTTCACTTTGGCAATCAAAGATTTTTGTAA

>cp32-13 CP001478.1:28736-30088 Borrelia burgdorferi CA-11.2a plasmid CA-11.2A

GTGAACTTATATCAAACAAAACTTTTTACAACACTACAAAAGGAATACAAAAATAAATATGGAGTTGACATATCACAATT

TGTAAAGCTAACAAATTCTTCAATTAATTTTGATAAGTTTGAAGAAAAACAGTTAACTTTAAAACAAAAAAATGTGATAA

AAAGCATTAAAAAGAATAATGAAAAAAAGATTATACTCAGCGGCGGCATAGCTAGCGGCAAAACGTATCTTGCATGCTAT

CTTTTTCTCAAAAGTTTAATTGAAAATAAAAAGTTATATTCTAGTGATACGAATAATTTTATTATTGGGAATTCACAACG

CTCAGTTGAAGTTAATGTTTTGGGACAATTTGAAAAGCTATGTAAACTTCTTAAAATTCCTTATATTCCAAGACATACAA

ATAATTCATATATTCTGATTGATTCACTACGTATTAATCTATATGGTGGAGATAAGGCAAGTGATTTTGAAAGATTTAGG

GGAAGTAATTCAGCACTTATTTTTGTTAATGAGGCTACAACTTTACACAAGCAAACTTTAGAGGAGGTCTTAAAAAGACT

AAGATGCGGGCAAGAAACTATTATTTTTGATACTAATCCTGATCATCCAGAACACTATTTTAAAACCGATTATATTGATA

ATATAGCGACATTTAAGACATATAATTTTACAACTTATGATAATGTTCTACTTAGTAAAGGATTTATCGAAACACAAGAA

AAACTCTATAAAGATATACCATCATATAAAGCAAGAGTTTTGCTAGGTGAGTGGATAGCAAGCACTGATTCAATTTTTAC

ACAAATAAATATTACTGATGATTATGTATTTACTAGTCCAATAGCATATTTAGACCCAGCATTTAGTGTTGGAGGAGATA

ACACTGCATTATGTGTTATGGAGCGAGTTGATGATAAGTATTATGCTTTTGTATTTCAAGACCAACGACCAGCCAATGAT

CCTTATATTATGAATATGGTAAAGACCGTTATAGAAAATTTCAATGTGCATACACTGTATTTAGAGGATAGAGATAATAC

AAAAGGTGCTGGTGGATTGACCCGCGAATACATCTTGCTAAGAAATAATATAAGCCAATATTTTAGAATTGTTCCAGTTA

AGCCAAAGTCTAATAAATTTAGCAGAATAACAACGTTAATTACGCCGTTTACTTATAAGAAACTTTACATTACAAAGTAC

AGTAGTTCTTCTGTATTTAATGATATTTATTCGTATAAGGGGGATAACAAAACCCATGATGATGCTCTTGATGCAATGTC

TGCAGCATATTTGATGTTGTCTTTAGGATATAGAGAGCGAAGTGTTCACTTTGGCAATCAAAGATTTTTGTAA

**Alignment of phage terminase large subunit**

cp32-12 GTGAACTTATATCAAACAAAACTTTTTACAACACTACAAAAGGAATACAAAAATAAATAT 60

cp32-9 GTGAACTTATATCAAACAAAACTTTTTACAACACTACAAAAGGAATACAAAAATAAATAT 60

cp32-13 GTGAACTTATATCAAACAAAACTTTTTACAACACTACAAAAGGAATACAAAAATAAATAT 60

cp32-2 GTGAACTTATATCAAACAAAACTTTTTACAACACTACAAAAGGAATACAAAAATAAATAT 60

cp32-10 GTGAACTTATATCAAACAAAACTTTTTACAACACTACAAAAGGAATACAAAAATAAATAT 60

cp32-11 GTGAACTTATATCAAACAAAACTTTTTACAACACTACAAAAGGAATACAAAAATAAATAT 60

cp32-8 GTGAACTTATATCAAACAAAACTTTTTACAACACTACAAAAGGAATACAAAAATAAATAT 60

cp32-1 GTGAACTTATATCAAACAAAACTTTTTACAACACTACAAAAGGAATACAAAAATAAATAT 60

cp32-5 GTGAACTTATATCAAACAAAACTTTTTACAACACTACAAAAGGAATACAAAAATAAATAT 60

cp32-6 GTGAACTTATATCAAACAAAACTTTTTACAACACTACAAAAGGAATATAAAAATAAATAT 60

cp32-3 GTGAACTTATATCAAACAAAACTTTTTACAACACTACAAAAGGAATATAAAAATAAATAT 60

cp32-4 GTGAACTTATATCAAACAAAACTTTTTACAACACTACAAAAGGAATATAAAAATAAATAT 60

cp32-7 GTGAACTTATATCAAACAAAACTTTTTACAACACTACAAAAGGAATATAAAAATAAATAT 60

*********************************************** ************

cp32-12 GGAGTTGACATATCACAATTTGTAAAGCTAACAAATTCTTCAATTAATTTTGATAAGTTT 120

cp32-9 GGAGTTGACATATCACAATTTGTAAAGCTAGCAAATTCTTCAATTAATTTTGATAAGTTT 120

cp32-13 GGAGTTGACATATCACAATTTGTAAAGCTAACAAATTCTTCAATTAATTTTGATAAGTTT 120

cp32-2 GGAGTTGATATATCACAATTTGTAAAGCTAACAAATTCTTCAATTAATTTTGATAAGTTT 120

cp32-10 GGAGTTGATATATCACAATTTGTAAAGCTAACAAATTCTTCAATTAATTTTGATAAGTTT 120

cp32-11 GGAGTTGATATATCACAATTTGTAAAGCTAACAAATTCTTCAATTAATTTTGATAAGTTT 120

cp32-8 GGAGTTGATATATCACAATTTGTAAAGCTAACAAATTCTTCAATTAATTTTGATAAGTTT 120

cp32-1 GGAGTTGATATATCACAATTTGTAAAGCTAACAAATTCTTCAATTAATTTTGATAAGTTT 120

cp32-5 GGAGTTGATATATCACAATTTGTAAAGCTAACAAATTCTTCAATTAATTTTGATAAGTTT 120

cp32-6 GGAGTTGACATATCACAATTTGTAAAGCTAACAAATTCTTCAATTAATTTTGATAAGTTT 120

cp32-3 GGAGTTGACATATCACAATTTGTAAAGCTAACAAATTCTTCAATTAATTTTGATAAGTTT 120

cp32-4 GGAGTTGACATATCACAATTTGTAAAGCTAACAAATTCTTCAATTAATTTTGATAAGTTT 120

cp32-7 GGAGTTGACATATCACAATTTGTAAAGCTAACAAATTCTTCAATTAATTTTGATAAGTTT 120

******** ********************* *****************************

cp32-12 GAAGAAAAACAGTTAACTTTAAAACAAAAAAATGTGATAAAAAGCATTAAAAAGAATAAT 180

cp32-9 GAAGAAAAACAGTTAACTTTAAAACAAAAAAATGTGATAAAAAGTATTAAAAAGAATAAT 180

cp32-13 GAAGAAAAACAGTTAACTTTAAAACAAAAAAATGTGATAAAAAGCATTAAAAAGAATAAT 180

cp32-2 GAAGAAGAACAGTTAACTTTAAAACAGAAAAATGTGATAAAAAGCATTGAAAAGAATAAT 180

cp32-10 GAAGAAGAACAGTTAACTTTAAAACAAAAAAATGTGATAAAAAGCATTAAAAAGAATAAT 180

cp32-11 GAAGAAGAACAGTTAACTTTAAAACAAAAAAATGTGATAAAAAGCATTAAAAAGAATAAT 180

cp32-8 GAAGAAGAACAGTTAACTTTAAAACAAAAAAATGTGATAAAAAGCATTAAAAAGAATAAT 180

cp32-1 GAAGAAGAACAGTTAACTTTAAAACAAAAAAATGTGATAAAAAGCATTAAAAAGAATAAT 180

cp32-5 GAAGAAGAACAGTTAACTTTAAAACAAAAAAATGTGATAAAAAGCATTAAAAAGAATAAT 180

cp32-6 GAAGAAAAACAGTTAACTTTAAAACAAAAAAATGTGATAAAAAGCATTCAAAAGAATAAT 180

cp32-3 GAAGAAAAACAGTTAACTTTAAAACAAAAAAATGTGATAAAAAGCATTAAAAAGAATAAT 180

cp32-4 GAAGAAAAACAGTTAACTTTAAAACAAAAAAATGTGATAAAAAGCATTCAAAAGAATAAT 180

cp32-7 GAAGAAAAACAGTTAACTTTAAAACAAAAAAATGTGATAAAAAGCATTCAAAAGAATAAT 180

****** ******************* ***************** *** ***********

cp32-12 GAAAAAAAGATTATACTCAGCGGCGGCATAGCTAGCGGCAAAACGTATCTTGCCTGTTAT 240

cp32-9 GAAAAAAAGATTATACTCAGCGGAGGCATAGCTAGTGGCAAAACGTATCTTGCATGTTAT 240

cp32-13 GAAAAAAAGATTATACTCAGCGGCGGCATAGCTAGCGGCAAAACGTATCTTGCATGCTAT 240

cp32-2 GAAAAGAAGATTATACTCAGTGGAGGTATAGCTAGCGGCAAAACGTATCTTGCATGTTAT 240

cp32-10 GAAAAAAAGATTATACTCAGCGGCGGCATAGCTAGCGGCAAAACGTATCTTGCATGTTAT 240

cp32-11 GAAAAGAAGATTATACTCAGTGGAGGTATAGCTAGTGGCAAAACGTATCTTGCATGTTAT 240

cp32-8 GAAAAGAAGATTATACTCAGCGGAGGCATAGCTAGTGGCAAAACGTATCTTGCATGTTAT 240

cp32-1 GAAAAGAAGATTATACTCAGCGGAGGCATAGCTAGTGGCAAAACGTATCTTGCATGTTAT 240

cp32-5 GAAAAGAAGATTATACTCAGCGGAGGCATAGCTAGTGGCAAAACGTATCTTGCATGTTAT 240

cp32-6 GAAAAGAAGATTATACTCAGCGGAGGCATAGCTAGTGGCAAAACGTATCTTGCATGTTAT 240

cp32-3 GAAAAGAAGATTATACTCAGCGGAGGCATAGCTAGTGGCAAAACGTATCTTGCATGTTAT 240

cp32-4 GAAAAGAAGATTATACTCAGCGGAGGCATAGCTAGTGGCAAAACGTATCTTGCATGTTAT 240

cp32-7 GAAAAGAAGATTATACTCAGCGGAGGCATAGCTAGTGGCAAAACGTATCTTGCATGTTAT 240

***** ************** ** ** ******** ***************** ** ***

cp32-12 CTTTTTCTAAAAAGTTTAATTGCAAATAAGAATTTATATTCTAGTGATACGAATAATTTC 300

cp32-9 CTTTTTCTAAAAAGTTTAATTGAAAATAAAAAGTTATACTCTAGTGATACTAATAATTTC 300

cp32-13 CTTTTTCTCAAAAGTTTAATTGAAAATAAAAAGTTATATTCTAGTGATACGAATAATTTT 300

cp32-2 CTTTTTCTAAAAAGTTTAATTGAAAATAAAAAGCTATATTCTAGCGATACGAATAATTTC 300

cp32-10 CTTTTTCTAAAAAGTTTAATTAAAAATAAAAAGTTATATTCTAGCGATACGAATAATTTT 300

cp32-11 CTTTTTCTAAAAAGTTTAATTAAAAATAAAAAGTTATACTCTAGTGATACTAATAATTTC 300

cp32-8 CTTTTTCTAAAAAGTTTAATTGAAAATAAAAAGTTATACTCTAGTGATACTAATAATTTC 300

cp32-1 CTTTTTCTAAAAAGTTTAATTGAAATTAAAAAGTTATACTCTAGTGATACTAATAATTTC 300

cp32-5 CTTTTTCTAAAAAGTTTAATTGAAAATAAAAAGTTATACTCTAGTGATACTAATAATTTC 300

cp32-6 CTTTTTCTAAAAAGTTTAATTGAAAATAAAAAGTTATACTCTAGTGATACTAATAATTTC 300

cp32-3 CTTTTTCTAAAAAGTTTAATTGAAAATAAAAAGTTATACTCTAGTGATACTAATAATTTT 300

cp32-4 CTTTTTCTAAAAAGTTTAATTGAAAATAAAAAGTTATACTCTAGTGATACTAATAATTTT 300

cp32-7 CTTTTTCTAAAAAGTTTAATTGAAAATAAAAAGTTATACTCTAGTGATACTAATAATTTT 300

******** ************ ** *** ** **** ***** ***** ********

cp32-12 ATTATTGGCAATTCGCAGCGCTCGGTTGAAGTTAATGTTTTGGGGCAATTTGAAAAGCTA 360

cp32-9 ATTATTGGGAATTCACAACGCTCAGTTGAAGTTAATGTTTTAGGACAATTTGAAAAGCTA 360

cp32-13 ATTATTGGGAATTCACAACGCTCAGTTGAAGTTAATGTTTTGGGACAATTTGAAAAGCTA 360

cp32-2 ATTATAGGGAATTCACAACGTTCAGTTGAAGTTAATGTTTTGGGACAATTTGAAAAGCTA 360

cp32-10 ATTATTGGGAATTCACAACGCTCAGTTGAAGTTAATGTTTTGGGACAATTTGAAAAGCTA 360

cp32-11 ATTATAGGGAATTCACAACGTTCAGTTGAAGTTAATGTTTTGGGGCAATTTGAAAAGCTA 360

cp32-8 ATTATAGGGAATTCACAACGTTCAGTTGAAGTTAATGTTTTGGGGCAATTTGAAAAGCTA 360

cp32-1 ATTATAGGGAATTCACAACGTTCAGTTGAAGTTAATGTTTTGGGGCAATTTGAAAAGCTA 360

cp32-5 ATTATAGGGAATTCACAACGTTCAGTTGAAGTTAATGTTTTGGGGCAATTTGAAAAGCTA 360

cp32-6 ATTATAGGGAATTCACAACGTTCAGTTGAAGTTAATGTTTTGGGACAATTTGAAAAGCTA 360

cp32-3 ATTATTGGGAATTCACAACGCTCAGTTGAAGTTAATGTTTTGGGACAATTTGAAAAGCTA 360

cp32-4 ATTATTGGGAATTCACAACGCTCAGTTGAAGTTAATGTTTTGGGACAATTTGAAAAGCTA 360

cp32-7 ATTATTGGGAATTCACAACGCTCAGTTGAAGTTAATGTTTTGGGACAATTTGAAAAGCTA 360

***** ** ***** ** ** ** ***************** ** ***************

cp32-12 TGCAAACGGCTTAAAATTCCTTATATTCCAAGACATACAAATAATTCATATATTCTGATT 420

cp32-9 TGTAAACTTCTTAAAATTCCTTATATCCCAAGACATACAAATAATTCATATATTCTGATT 420

cp32-13 TGTAAACTTCTTAAAATTCCTTATATTCCAAGACATACAAATAATTCATATATTCTGATT 420

cp32-2 TGTAAACTTCTTAAAATTCCTTATATTCCAAGACATACAAATAATTCATATATTCTGATT 420

cp32-10 TGTAAACTTCTTAAAATTCCTTATATTCCAAGACATACAAATAATTCATATATTCTTATT 420

cp32-11 TGTAAACTTCTTAAAATTCCTTATATTCCAAGACATACAAATAATTCATATATTCTGATT 420

cp32-8 TGTAAACTTCTTAAAATTCCTTATATTCCAAGACATACAAATAATTCATATATTCTGATT 420

cp32-1 TGTAAACTTCTTAAAATTCCTTATATTCCAAGACATACAAATAATTCATATATTCTGATT 420

cp32-5 TGTAAACTTCTTAAAATTCCTTATATTCCAAGACATACAAATAATTCATATATTCTGATT 420

cp32-6 TGTAAACTTCTTAAAATTCCTTATATCCCAAGACATACAAATAATTCATATATTCTGATT 420

cp32-3 TGTAAACTTCTTAAAATTCCTTATATCCCAAGACATACAAATAATTCATATATTCTGATT 420

cp32-4 TGTAAACTTCTTAAAATTCCTTATATCCCAAGACATACAAATAATTCATATATTCTGATT 420

cp32-7 TGTAAACTTCTTAAAATTCCTTATATCCCAAGACATACAAATAATTCATATATTCTGATT 420

** **** ***************** ***************************** ***

cp32-12 GATTCACTACGTATTAATCTATATGGTGGAGATAAGGCAAGTGATTTTGAAAGATTTAGG 480

cp32-9 GATTCACTACGTATTAATCTATATGGTGGAGATAAGGCAAGTGATTTTGAAAGATTTAGG 480

cp32-13 GATTCACTACGTATTAATCTATATGGTGGAGATAAGGCAAGTGATTTTGAAAGATTTAGG 480

cp32-2 GATTCACTGCGAATTAATCTATATGGTGGAGATAAGGCAAGTGTTTTTGAAAGATTTAGG 480

cp32-10 GATTCACTGAGAATTAATCTATATGGAGGAGATAAGGCAAGTGATTTTGAAAGATTTAGG 480

cp32-11 GATTCACTGCGAATTAATCTATATGGTGGAGATAAGGCAAGTGATTTTGAAAGATTTAGG 480

cp32-8 GATTCACTACGTATTAATCTATATGGAGGAGATAAGGCAAGTGATTTTGAAAGATTTAGG 480

cp32-1 GATTCACTACGTATTAATCTATATGGAGGAGATAAGGCAAGTGATTTTGAAAGATTTAGG 480

cp32-5 GATTCACTACGTATTAATCTATATGGAGGAGATAAGGCAAGTGATTTTGAAAGATTTAGG 480

cp32-6 GATTCACTACGTATTAATCTATATGGTGGAGATAAGGCAAGTGATTTTGAAAGATTTAGG 480

cp32-3 GATTCACTACGTATTAATCTATATGGTGGAGATAAGGCAAGTGATTTTGAAAGATTTAGG 480

cp32-4 GATTCACTACGTATTAATCTATATGGTGGAGATAAGGCAAGTGATTTTGAAAGATTTAGG 480

cp32-7 GATTCACTACGTATTAATCTATATGGTGGAGATAAGGCAAGTGATTTTGAAAGATTTAGG 480

******** * ************** **************** ****************

cp32-12 GGAAGTAATTCAGCGCTTATTTTTGTTAATGAGGCTACAACTTTACACAGGCAAACTTTA 540

cp32-9 GGAAGTAATTCAGCACTTATTTTTGTGAATGAGGCTACAACTTTACACAAGCAAACTTTA 540

cp32-13 GGAAGTAATTCAGCACTTATTTTTGTTAATGAGGCTACAACTTTACACAAGCAAACTTTA 540

cp32-2 GGAAGTAATTCAGCACTTATTTTTGTGAATGAGGCTACAACTTTACACAAGCAAACTTTA 540

cp32-10 GGAAGTAATTCGGCACTTATTTTTGTTAATGAGGCTACAACTTTACACAAGCAAACTTTA 540

cp32-11 GGAAGTAATTCAGCACTTATTTTTGTTAATGAGGCTACAACTTTACACAAGCAAACTTTA 540

cp32-8 GGAAGTAATTCGGCACTTATTTTTGTTAATGAGGCTACAACTTTACACAAGCAAACTTTA 540

cp32-1 GGAAGTAATTCGGCACTTATTTTTGTTAATGAGGCTACAACTTTACACAAGCAAACTTTA 540

cp32-5 GGAAGTAATTCGGCACTTATTTTTGTTAATGAGGCTACAACTTTACACAAGCAAACTTTA 540

cp32-6 GGAAGTAATTCGGCACTTATTTTTGTTAATGAGGCTACAACTTTACACAAGCAAACTTTA 540

cp32-3 GGAAGTAATTCGGCACTTATTTTTGTTAATGAGGCTACAACTTTACACAAGCAAACTTTA 540

cp32-4 GGAAGTAATTCGGCACTTATTTTTGTTAATGAGGCTACAACTTTACACAAGCAAACTTTA 540

cp32-7 GGAAGTAATTCGGCACTTATTTTTGTTAATGAGGCTACAACTTTACACAAGCAAACTTTA 540

*********** ** *********** ********************** **********

cp32-12 GAGGAAGTCTTAAAAAGACTAAGGTGTGGGCAAGAAACTATTATTTTTGATACTAACCCC 600

cp32-9 GAGGAGGTCTTAAAAAGACTAAGATGCGGGCAAGAAACTATTATTTTTGATACTAATCCT 600

cp32-13 GAGGAGGTCTTAAAAAGACTAAGATGCGGGCAAGAAACTATTATTTTTGATACTAATCCT 600

cp32-2 GAGGAGGTCTTAAAAAGACTAAGATGCGGGCAAGAAACTATTATTTTTGATACTAATCCT 600

cp32-10 GAGGAGGTCTTAAAAAGACTAAGATGCGGGCAAGAAACTATTATTTTTGATACTAATCCC 600

cp32-11 GAGGAGGTCTTAAAAAGACTAAGATGCGGGCAAGAAACTATTATTTTTGATACTAATCCC 600

cp32-8 GAGGAAGTCTTAAAAAGACTAAGATGCGGGCAAGAAACTATTATTTTTGATACTAATCCT 600

cp32-1 GAGGAAGTCTTAAAAAGACTAAGATGCGGGCAAGAAACTATTATTTTTGATACTAATCCT 600

cp32-5 GAGGAAGTCTTAAAAAGACTAAGATGCGGGCAAGAAACTATTATTTTTGATACTAATCCT 600

cp32-6 GAGGAAGTCTTAAAAAGACTAAGATGCGGGCAAGAAACTATTATTTTTGATACTAATCCT 600

cp32-3 GAGGAGGTCTTAAAAAGACTAAGATGCGGGCAAGAAACTATTATTTTTGATACTAATCCT 600

cp32-4 GAGGAAGTCTTAAAAAGACTAAGATGCGGGCAAGAAACTATTATTTTTGATACTAATCCT 600

cp32-7 GAGGAAGTCTTAAAAAGACTAAGATGCGGGCAAGAAACTATTATTTTTGATACTAATCCT 600

***** ***************** ** ***************************** **

cp32-12 GATCATCCGGAACACTATTTTAAAACCGATTATATTGATAATATAGCAACTTTTAAGACA 660

cp32-9 GATCATCCAGAACACTATTTTAAAACCGATTATATTGATAATATAGCGACATTTAAGACA 660

cp32-13 GATCATCCAGAACACTATTTTAAAACCGATTATATTGATAATATAGCGACATTTAAGACA 660

cp32-2 GATCATCCAGAACACTATTTTAAAACCGATTATATTGATAATATAGCGACCTTTAAGACA 660

cp32-10 GATCATCCAGAACACTATTTTAAAACCGATTATATTGATAATATAGCGACATTTAAGACA 660

cp32-11 GATCATCCAGAACACTATTTTAAAACCGATTATATTGATAATATAGCGACCTTTAAGACA 660

cp32-8 GATCATCCAGAACACTATTTTAAAACCGATTATATTGATAATATAGCGACCTTTAAGACA 660

cp32-1 GATCATCCAGAACACTATTTTAAAACCGATTATATTGATAATATAGCGACCTTTAAGACA 660

cp32-5 GATCATCCAGAACACTATTTTAAAACCGATTATATTGATAATATAGCGACCTTTAAGACA 660

cp32-6 GATCATCCAGAACACTATTTTAAAACCGATTATATTGATAATATAGCGACCTTTAAGACA 660

cp32-3 GATCATCCAGAACACTATTTTAAAACCGATTATATTGATAATATAGCGACCTTTAAGACA 660

cp32-4 GATCATCCAGAACACTATTTTAAAACCGATTATATTGATAATATAGCGACCTTTAAGACA 660

cp32-7 GATCATCCAGAACACTATTTTAAAACCGATTATATTGATAATATAGCGACCTTTAAGACA 660

******** ************************************** ** *********

cp32-12 TATAATTTTACAACTTATGATAATGTTCTACTTAGTAAAGGATTTATCGAAACACAAGAA 720

cp32-9 TATAATTTTACAACTTATGATAATGTGCTACTTAGTAAAGGATTTATCGAAACACAAGAA 720

cp32-13 TATAATTTTACAACTTATGATAATGTTCTACTTAGTAAAGGATTTATCGAAACACAAGAA 720

cp32-2 TATAATTTCACAACTTATGATAATGTGCTACTTAGTAAAGGATTTGTCGAAACACAAGAA 720

cp32-10 TATAATTTTACAACTTATGATAATGTGCTACTTAGTAAAGGATTTGTCGAAACACAAGAA 720

cp32-11 TATAATTTTACAACTTATGATAATGTGCTACTTAGTAAAGGATTTGTCGAAACACAAGAA 720

cp32-8 TATAAGTTTACAACTTATGATAATGTGCTACTTAGTAAAGGATTTGTCGAAACACAAGAA 720

cp32-1 TATAAGTTTACAACTTATGATAATGTGCTACTTAGTAAAGGATTTGTCGAAACACAAGAA 720

cp32-5 TATAAGTTTACAACTTATGATAATGTGCTACTTAGTAAAGGATTTGTCGAAACACAAGAA 720

cp32-6 TATAATTTTACAACTTATGATAATGTGCTACTTAGTAAAGGATTTGTCGAAACACAAGAA 720

cp32-3 TATAATTTTACAACTTATGATAATGTGCTACTTAGTAAAGGATTTGTCGAAACACAAGAA 720

cp32-4 TATAAGTTTACAACTTATGATAATGTGCTACTTAGTAAAGGATTTGTCGAAACACAAGAA 720

cp32-7 TATAATTTTACAACTTATGATAATGTGCTACTTAGTAAAGGATTTGTCGAAACACAAGAA 720

***** ** ***************** ****************** **************

**cp32-12 AAACTCTATAAAGATATACCATCATATAAAGCAAGAGTTTTGCTAGGTGAGTGGATAGCA 780**

**cp32-9 AAACTCTATAAAGATATACCATCATATAAAGCAAGAGTTTTGCTAGGTGAGTGGATAGCA 780**

**cp32-13 AAACTCTATAAAGATATACCATCATATAAAGCAAGAGTTTTGCTAGGTGAGTGGATAGCA 780**

**cp32-2 AAGCTATATAAAGATATACCATCATATAAAGCAAGAGTTTTGCTAGGTGAATGGATAGCA 780**

**cp32-10 AAGCTATATAAAGATATACCATCATATAAAGCAAGAGTTTTGCTAGGTGAGTGGATAGCA 780**

**cp32-11 AAGCTATATAAAGATATACCATCATATAAAGCAAGAGTTTTGCTAGGTGAGTGGATAGCA 780**

**cp32-8 AAGCTATATAAAGATATACCATCATATAAAGCAAGAGTTTTGTTAGGTGAGTGGATAGCA 780**

**cp32-1 AAGCTATATAAAGATATACCATCATATAAAGCAAGAGTTTTGTTAGGTGAGTGGATAGCA 780**

**cp32-5 AAGCTATATAAAGATATACCATCATATAAAGCAAGAGTTTTGTTAGGTGAGTGGATAGCA 780**

**cp32-6 AAGCTATATAAAGATATACCATCATATAAAGCAAGAGTTTTGTTAGGTGAGTGGATAGCA 780**

**cp32-3 AAGCTATATAAAGATATACCATCATATAAAGCAAGAGTTTTGTTAGGTGAGTGGATAGCA 780**

**cp32-4 AAGCTATATAAAGATATACCATCATATAAAGCAAGAGTTTTGTTAGGTGAGTGGATAGCA 780**

**cp32-7 AAGCTATATAAAGATATACCATCATATAAAGCAAGAGTTTTGTTAGGTGAGTGGATAGCA 780**

**** ** ************************************ ******* ***********

**cp32-12 AGCACTGATTCAATTTTTACACAAATAAATATTACTGATGATTATGTATTTACTAGCCCG 840**

**cp32-9 AGCACTGATTCAATTTTTACACAAATAAATATTACTGATGATTATATATTTACTAGCCCG 840**

**cp32-13 AGCACTGATTCAATTTTTACACAAATAAATATTACTGATGATTATGTATTTACTAGTCCA 840**

**cp32-2 AGCACCGATTCAATTTTTACACAAATAAATATTACTGATGATTATGTATTTACTAGCCCG 840**

**cp32-10 AGCACTGATTCAATTTTTACACAAATAAATATTACTGATGATTATGTATTTACTAGCCCG 840**

**cp32-11 AGCACTGATTCAATTTTTACACAAATAAATATTACTGATGATTATGTATTTACTAGCCCG 840**

**cp32-8 AGCACTGATTCAATTTTTACACAAATAAATATTACTGATGATTATGTATTTACTAGCCCG 840**

**cp32-1 AGCACTGATTCAATTTTTACACAAATAAATATTACTGATGATTATGTATTTACTAGCCCG 840**

**cp32-5 AGCACTGATTCAATTTTTACACAAATAAATATTACTGATGATTATGTATTTACTAGCCCG 840**

**cp32-6 AGCACTGATTCAATTTTTACACAAATAAATATTACTGATGATTATGTATTTACTAGCCCA 840**

**cp32-3 AGCACCGATTCAATTTTTACACAAATAAATATTACTGATGATTATGTATTTACTAGCCCG 840**

**cp32-4 AGCACTGATTCAATTTTTACACAAATAAATATTACTGATGATTATGTATTTACTAGCCCG 840**

**cp32-7 AGCACTGATTCAATTTTTACACAAATAAATATTACTGATGATTATGTATTTACTAGCCCG 840**

******* *************************************** ********** ****

**cp32-12 ATAGCATATTTAGACCCAGCATTTAGTGTTGGCGGGGATAACACTGCATTATGTGTTATG 900**

**cp32-9 ATAGCATATTTAGACCCAGCATTTAGTGTTGGAGGGGATAACACTGCATTATGTGTTATG 900**

**cp32-13 ATAGCATATTTAGACCCAGCATTTAGTGTTGGAGGAGATAACACTGCATTATGTGTTATG 900**

**cp32-2 ATAGCATATTTAGACCCAGCATTTAGTGTTGGAGGGGATAACACTGCATTATGTGTTATG 900**

**cp32-10 ATAGCATATTTAGACCCAGCATTTAGTGTTGGCGGGGATAACACTGCATTATGTGTTATG 900**

**cp32-11 ATAGCATATTTAGACCCAGCATTTAGTGTTGGCGGGGATAACACTGCATTATGTGTTATG 900**

**cp32-8 ATAGCATATTTAGACCCAGCATTTAGTGTTGGAGGGGATAACACTGCATTATGTGTTATG 900**

**cp32-1 ATAGCATATTTAGACCCAGCATTTAGTGTTGGCGGGGATAACACTGCATTATGTGTTATG 900**

**cp32-5 ATAGCATATTTAGACCCAGCATTTAGTGTTGGCGGGGATAACACTGCATTATGTGTTATG 900**

**cp32-6 ATAGCATATTTAGACCCAGCATTTAGTGTTGGAGGAGATAACACTGCATTATGTGTTATG 900**

**cp32-3 ATAGCATATTTAGACCCAGCATTTAGTGTTGGCGGGGATAACACTGCATTATGTGTTATG 900**

**cp32-4 ATAGCATATTTAGACCCAGCATTTAGTGTTGGCGGGGATAACACTGCATTATGTGTTATG 900**

**cp32-7 ATAGCATATTTAGACCCAGCATTTAGTGTTGGCGGGGATAACACTGCATTATGTGTTATG 900**

********************************** ** **************************

**cp32-12 GAGCGAGTTGATGATAAGTATTATGCTTTTGTATTTCAAGACCAACGACCAGCTAATGAT 960**

**cp32-9 GAGCGAGTTGATGATAAGTATTATGCTTTTGTATTTCAAGACCAACGACCAGCCAATGAT 960**

**cp32-13 GAGCGAGTTGATGATAAGTATTATGCTTTTGTATTTCAAGACCAACGACCAGCCAATGAT 960**

**cp32-2 GAGCGAATTGATGATAAGTATTATGCTTTTGTATTTCAAGACCAACGACCAGCCAATGAT 960**

**cp32-10 GAGCGAGTTGATGATAAGTATTATGCTTTTGTATTTCAAGACCAAAGACCAGCTAATGAT 960**

**cp32-11 GAGCGAGTTGATGATAAGTATTATGCTTTTGTATTTCAAGACCAAAGACCAGCTAATGAT 960**

**cp32-8 GAGCGAGTTGATGATAAGTATTATGCTTTTGTATTTCAAGACCAAAGACCAGCTAATGAT 960**

**cp32-1 GAGCGAGTTGATGATAAGTATTATGCTTTTGTATTTCAAGACCAAAGACCAGCTAATGAT 960**

**cp32-5 GAGCGAGTTGATGATAAGTATTATGCTTTTGTATTTCAAGACCAAAGACCAGCTAATGAT 960**

**cp32-6 GAGCGAGTTGATGATAAGTATTATGCTTTTGTATTTCAAGACCAACGACCAGCCAATGAT 960**

**cp32-3 GAGCGAGTTGATGATAAGTATTATGCTTTTGTATTTCAAGACCAAAGACCAGCTAATGAT 960**

**cp32-4 GAGCGAGTTGATGATAAGTATTATGCTTTTGTATTTCAAGACCAAAGACCAGCTAATGAT 960**

**cp32-7 GAGCGAGTTGATGATAAGTATTATGCTTTTGTATTTCAAGACCAAAGACCAGCTAATGAT 960**

******** ************************************** ******* ********

cp32-12 CCTTATATTATGAATATGGTAAAGACCGTTATAGAAAATTTCAATGTGCATACACTGTAT 1020

cp32-9 CCTTATATTATGAATATGGTAAAGACCGTTATAGAAAATTTCAATGTGCATACACTGTAT 1020

cp32-13 CCTTATATTATGAATATGGTAAAGACCGTTATAGAAAATTTCAATGTGCATACACTGTAT 1020

cp32-2 CCTTATATTATGAATATGGTAAAGACCGTTATAGAAAATTTCAATGTGCATACACTGTAT 1020

cp32-10 CCTTATATTATGAATATGGTAAAGACTGTTATAGAAAATTTCAATGTGCATACACTGTAT 1020

cp32-11 CCTTATATTATGAATATGGTAAAGACCGTTATAGAAAATTTCAATGTGCATACACTGTAT 1020

cp32-8 CCTTATATTATGAATATGGTAAAGACTGTTATAGAAAATTTCAATGTGCATACACTGTAT 1020

cp32-1 CCTTATATTATGAATATGGTAAAGACTGTTATAGAAAATTTCAATGTGCATACACTGTAT 1020

cp32-5 CCTTATATTATGAATATGGTAAAGACTGTTATAGAAAATTTCAATGTGCATACACTGTAT 1020

cp32-6 CCTTATATTATGAATATCGTAAAGACTGTTATAGAAAATTTCAATGTGCATACACTGTAT 1020

cp32-3 CCTTATATTATGAATATGGTAAAGACTGTTATAGAAAATTTCAATGTGCATACACTGTAT 1020

cp32-4 CCTTATATTATGAATATGGTAAAGACTGTTATAGAAAATTTCAATGTGCATACACTGTAT 1020

cp32-7 CCTTATATTATGAATATGGTAAAGACTGTTATAGAAAATTTCAATGTGCATACACTGTAT 1020

***************** ******** *********************************

cp32-12 TTAGAGGATAGAGATAATACAAAAGGTGCTGGTGGATTGACCCGTGAATACATCTTGCTA 1080

cp32-9 TTAGAGGATAGAGATAATACAAAAGGTGCTGGTGGATTGACCCGCGAATACATCTTGCTA 1080

cp32-13 TTAGAGGATAGAGATAATACAAAAGGTGCTGGTGGATTGACCCGCGAATACATCTTGCTA 1080

cp32-2 TTAGAGGATAGAGATAATACAAAAGGTGCTGGTGGATTGACCCGTGAATACATCTTGCTA 1080

cp32-10 TTAGAGGATAGAGATAATACAAAAGGTGCTGGTGGATTGACCCGTGAATACATCTTGCTA 1080

cp32-11 TTAGAGGATAGAGATAATACAAAAGGTGCTGGTGGATTGACCCGTGAATACATCTTGCTA 1080

cp32-8 TTAGAGGATAGAGATAATACAAAAGGTGCTGGTGGATTGACCCGTGAATACATCTTGCTA 1080

cp32-1 TTAGAGGATAGAGATAATACAAAAGGTGCTGGTGGATTGACCCGTGAATACATCTTGCTA 1080

cp32-5 TTAGAGGATAGAGATAATACAAAAGGTGCTGGTGGATTGACCCGTGAATACATCTTGCTA 1080

cp32-6 TTAGAGGATAGAGATAATACAAAAGGTGCTGGTGGATTGACCCGTGAATACATCTTACTA 1080

cp32-3 TTAGAGGATAGAGATAATACAAAAGGTGCTGGTGGATTGACCCGTGAATACATCTTGCTA 1080

cp32-4 TTAGAGGATAGAGATAATACAAAAGGTGCTGGTGGATTGACCCGTGAATACATCTTGCTA 1080

cp32-7 TTAGAGGATAGAGATAATACAAAAGGTGCTGGTGGATTGACCCGTGAATACATCTTGCTA 1080

******************************************** *********** ***

cp32-12 AGAAATAATATAAGCCAATATTTTAGAATTGTTCCAGTTAAGCCAAAGTCTAATAAATTT 1140

cp32-9 AGAAATAATATAAGCCAATATTTTAGAATTGTTCCAGTTAAGCCAAAGTCTAATAAATTT 1140

cp32-13 AGAAATAATATAAGCCAATATTTTAGAATTGTTCCAGTTAAGCCAAAGTCTAATAAATTT 1140

cp32-2 AGAAATAATACGAGTCAATATTTTAGAATTGTTCCAGTTAAGCCAAAGTCTAATAAATTT 1140

cp32-10 AGAAGTAATATAAGCCAATATTTTAGAATTGTTCCAGTTAAGCCAAAGTCTAATAAATTT 1140

cp32-11 AGAAGTAATATAAGCCAATATTTTAGAATTGTTCCAGTTAAGCCAAAGTCTAATAAATTT 1140

cp32-8 AGAAGTAATATAAGCCAATATTTTAGAATTGTTCCAGTTAAGCCAAAGTCTAATAAATTT 1140

cp32-1 AGAAGTAATATAAGCCAATATTTTAGAATTGTTCCAGTTAAGCCAAAGTCTAATAAATTT 1140

cp32-5 AGAAGTAATATAAGCCAATATTTTAGAATTGTTCCAGTTAAGCCAAAGTCTAATAAATTT 1140

cp32-6 AGAAGTAATATAAGCCAATATTTTAGAATTGTTCCAGTTAAGCCAAAGTCTAATAAATTT 1140

cp32-3 AGAAGTAATATAAGCCAATATTTTAGAATTGTTCCAGTTAAGCCAAAGTCTAATAAATTT 1140

cp32-4 AGAAGTAATATAAGCCAATATTTTAGAATTGTTCCAGTTAAGCCAAAGTCTAATAAATTT 1140

cp32-7 AGAAGTAATATAAGCCAATATTTTAGAATTGTTCCAGTTAAGCCAAAGTCTAATAAATTT 1140

**** ***** ** *********************************************

cp32-12 AGCAGAATAACAACGTTAATTACGCCGTTTACTTATAAGAAACTTTACATTACAAAGTAC 1200

cp32-9 AGCAGAATAACAACGTTAATTACGCCGTTTACTTACAAAAAACTTTATATTACAAAGTAC 1200

cp32-13 AGCAGAATAACAACGTTAATTACGCCGTTTACTTATAAGAAACTTTACATTACAAAGTAC 1200

cp32-2 AGCAGAATAACAACGTTAATTACGCCGTTTACTTATAAGAAACTTTACATTACAAAGTAC 1200

cp32-10 AGCAGAATAACAACGTTAATTACGCCGTTTACTTACAAAAAACTTTATATTACAAAGTAC 1200

cp32-11 AGCAGAATAACAACGTTAATTACGCCGTTTACTTACAAAAAACTTTATATTACAAAGTAC 1200

cp32-8 AGCAGAATAACAACGTTAATTACGCCGTTTACTTACAAAAAACTTTATATTACAAAGTAC 1200

cp32-1 AGCAGAATAACAACGTTAATTACGCCGTTTACTTACAAAAAACTTTATATTACAAAGTAC 1200

cp32-5 AGCAGAATAACAACGTTAATTACGCCGTTTACTTACAAAAAACTTTATATTACAAAGTAC 1200

cp32-6 AGCAGAATAACAACGTTAATTACGCCGTTTACTTACAAAAAACTTTACATTACAAAGTAC 1200

cp32-3 AGCAGAATAACAACGTTAATTACGCCGTTTACTTACAAAAAACTTTATATTACAAAGTAC 1200

cp32-4 AGCAGAATAACAACGTTAATTACGCCGTTTACTTACAAAAAACTTTATATTACAAAGTAC 1200

cp32-7 AGCAGAATAACAACGTTAATTACGCCGTTTACTTACAAAAAACTTTATATTACAAAGTAC 1200

*********************************** ** ******** ************

cp32-12 AGTAGTTCTTCTGTATTTAATGATATTTATTCGTATAAGGGGGATAACAAAACCCATGAT 1260

cp32-9 AGTAGTTCTTCTGTATTTAATGATATTTATTCGTATAAGGGGGATAGCAAAACCCATGAT 1260

cp32-13 AGTAGTTCTTCTGTATTTAATGATATTTATTCGTATAAGGGGGATAACAAAACCCATGAT 1260

cp32-2 AGTAGTTCTTCTGTATTTAATGATATTTATTCGTATAAGGGGGATAACAAAACCCATGAT 1260

cp32-10 AGTAGTTCTTCTGTATTTAATGATATTTATTCGTATAAGGGGGATAGCAAAACCCATGAT 1260

cp32-11 AGTAGTTCTTCTGTATTTAATGATATTTATTCGTATAAGGGGGATAGCAAAACCCATGAT 1260

cp32-8 AGTAGTTCTTCCGTATTTAATGATATTTATTCGTATAAGGGGGATAATAAAACCCATGAT 1260

cp32-1 AGTAGTTCTTCCGTATTTAATGATATTTATTCGTATAAGGGGGATAATAAAACCCATGAT 1260

cp32-5 AGTAGTTCTTCCGTATTTAATGATATTTATTCGTATAAGGGGGATAATAAAACCCATGAT 1260

cp32-6 AGTAGTTCTTCTGTATTTAATGATATTTATTCGTATAAGGGGGATAATAAAACCCATGAT 1260

cp32-3 AGTAGTTCTTCCGTATTTAATGATATTTATTCGTATAAGGGGGATAATAAAACCCATGAT 1260

cp32-4 AGTAGTTCTTCTGTATTTAATGATATTTATTCGTATAAGGGGGATAGCAAAACCCATGAT 1260

cp32-7 AGTAGTTCTTCTGTATTTAATGATATTTATTCGTATAAGGGGGATAGCAAAACCCATGAT 1260

*********** ********************************** ************

cp32-12 GATGCTCTTGATGCAATGTCTGCAGCATATTTGATGTTGTCTTTAGGATATAGAGAGCGA 1320

cp32-9 GATGCTCTTGATGCAATATCTGCAGCATATTTGATGTTGTCTTTAGGATATAGAGAGCGA 1320

cp32-13 GATGCTCTTGATGCAATGTCTGCAGCATATTTGATGTTGTCTTTAGGATATAGAGAGCGA 1320

cp32-2 GATGCTCTTGATGCAATGTCTGCAGCATATTTGATGTTGTCTTTAGGATATAGAGAGCGA 1320

cp32-10 GATGCTCTTGATGCAATGTCTGCAGCATATTTGATGTTGTCTTTAGGATATAGAGAGCGA 1320

cp32-11 GATGCTCTTGATGCAATGTCTGCAGCATATTTGATGTTGTCTTTAGGATATAGAGAGCGA 1320

cp32-8 GACGCTCTTGATGCAATATCTGCAGCATATTTGATGTTGTCTTTAGGATATAGAGAGCGA 1320

cp32-1 GACGCTCTTGATGCAATATCTGCAGCATATTTGATGTTGTCTTTAGGATATAGAGAGCGA 1320

cp32-5 GACGCTCTTGATGCAATATCTGCAGCATATTTGATGTTGTCTTTAGGATATAGAGAGCGA 1320

cp32-6 GATGCTCTTGATGCAATGTCTGCAGCATATTTGATGTTGTCTTTAGGATATAGAGAGCGA 1320

cp32-3 GATGCTCTTGATGCAATGTCTGCAGCATATTTGATGTTGTCTTTAGGATATAGAGAGCGA 1320

cp32-4 GATGCTCTTGATGCAATGTCTGCAGCATATTTGATGTTGTCTTTAGGATATAGAGAGCGA 1320

cp32-7 GATGCTCTTGATGCAATGTCTGCAGCATATTTGATGTTGTCTTTAGGATATAGAGAGCGA 1320

** ************** ******************************************

cp32-12 AGTGTTCACTTTGGCAACCAAAGATTTTTGTAA 1353

cp32-9 AGTGTTCACTTTGGCAATCAAAGATTTTTGTAA 1353

cp32-13 AGTGTTCACTTTGGCAATCAAAGATTTTTGTAA 1353

cp32-2 AGTGTTCACTTTGGCAATCAAAGATTTTTGTAA 1353

cp32-10 AGTGTTCACTTTGGCAATCAAAGATTTTTGTAA 1353

cp32-11 AGTGTTCACTTTGGCAATCAAAGATTTTTGTAA 1353

cp32-8 AGTGTTCACTTTGGCAATCAAAGATTTTTGTAA 1353

cp32-1 AGTGTTCACTTTGGCAATCAAAGATTTTTGTAA 1353

cp32-5 AGTGTTCACTTTGGCAATCAAAGATTTTTGTAA 1353

cp32-6 AGTGTTCACTTTGGCAATCAAAGATTTTTGTAA 1353

cp32-3 AGTGTTCACTTTGGCAATCAAAGATTTTTGTAA 1353

cp32-4 AGTGTTCACTTTGGCAATCAAAGATTTTTGTAA 1353

cp32-7 AGTGTTCACTTTGGCAATCAAAGATTTTTGTAA 1353

***************** ***************

**Supplementary information 4**:

To ensure the specificity of the primer/probe set, BLAST analysis using sequences submitted to GenBank was performed. All hits with e-value < 0.01 were Lyme *Borrelia* species dominated by *B. burgdorferi* with multiple hits of the following *Borrelia* strains: *B. mayonii*, *B. garinii*, *B. afzelii*, *B. bisettii*, and *B. valasiana* (BLAST was carried out on the 5^th^ October 2020). According to the discovery of widespread presence of the *terL*-bearing cp32 plasmids, we predicted that the Ter-qPCR would show positive PCR results against Lyme *Borrelia* species except *B. spielmanii*. Indeed, ‘*In silico’* PCR (http://insilico.ehu.es/PCR/) revealed PCR product of the correct size from the plasmid fractions of *B. burgdorferi*, *B. mayonii*, *B. garinii*, *B. afzelii*, *B. bisettii*, *B. valasiana, B. bavariensis, B. finlandensis*. No PCR products were amplified from *B. spielmanii*. Positive PCR results were also obtained from all the LD *Borrelia* strains listed in Table 2, except *B. spielmanii*. No positive PCR results were ever observed from RF *Borrelia* strains including *B. miyamotoi* (Table 1) and other bacterial strains that have been used in the lab, including *Clostridium difficile*, *Clostridium perfringens*, *Escherichia coli*, *Pseudomonas aeruginosa*, *Streptococcus pneumoniae*, *Staphylococcus aureus*, *Burkholderia thailandensis*, *Burkholderia pseudomallei*, *Haemophilus influenzae* and *Salmonella enterica*.

To rule out the possibility of unspecific amplification with human DNA, UCSC In-Silico PCR (<https://genome.ucsc.edu/cgi-bin/hgPcr>) was conducted against human DNA. No PCR product was observed. In addition, commercial human female and male DNA and total DNA extracted from commercial HWB also didn’t display any positive Ter-qPCRs, while reference qPCR targeting human housekeeping genes RNase P (TaqMan™ Copy Number Reference Assay, Catalog number: 4403326) produced positive results. This confirmed that the Ter-qPCR did not amplify human DNA.

**Supplementary information 5: Estimation of LOD of the Ter-qPCR using diluted Ter-plasmid DNA.** The Ter-plasmid DNA was diluted from 1000 copies to 100, 80, 60, 40, 20, 10, 5, and 1 copies/PCR with a human DNA background of 125 ng per PCR. Ten replicates were used for each dilution. The LOD was calculated as 22 copies according to Probit analysis in SPSS with 95% probability. Positive qPCR has typical PCR amplification curve with a Cq<38.

| **Copy number/PCR** | **Number of replicates** | **Number of PCR positive replicates (% of positive)** |
| --- | --- | --- |
| 1000 | 10 | 10 (100%) |
| 100 | 10 | 10 (100%) |
| 80 | 10 | 10 (100%) |
| 60 | 10 | 10 (100%) |
| 40 | 10 | 10 (100%) |
| 20 | 10 | 9 (90%) |
| 10 | 10 | 7 (70%) |
| 5 | 10 | 4 (40%) |
| 1 | 10 | 2 (20%) |
